# Synthesis and Antiproliferative Effect of New Alkyne-Tethered Vindoline Hybrids Containing Pharmacophoric Fragments

**DOI:** 10.3390/ijms25137428

**Published:** 2024-07-06

**Authors:** Etelka Ferenczi, Péter Keglevich, Bizhar Ahmed Tayeb, Renáta Minorics, Dávid Papp, Gitta Schlosser, István Zupkó, László Hazai, Antal Csámpai

**Affiliations:** 1 Department of Organic Chemistry, Eötvös Loránd University (ELTE), Pázmány P. sétány 1/A, H-1117 Budapest, Hungary; ferenczie@student.elte.hu; 2 Hevesy György PhD School of Chemistry, Pázmány P. sétány 1/A, H-1117 Budapest, Hungary; david.papp@ttk.elte.hu; 3 Department of Organic Chemistry and Technology, Faculty of Chemical Technology and Biotechnology, Budapest University of Technology and Economics, Műegyetem rkp. 3, H-1111 Budapest, Hungary; keglevich.peter@vbk.bme.hu (P.K.); hazai.laszlo@vbk.bme.hu (L.H.); 4 Institute of Pharmacodynamics and Biopharmacy, University of Szeged, Eötvös u. 6, H-6720 Szeged, Hungary; drbat25@gmail.com (B.A.T.); kanizsaine.minorics.renata@szte.hu (R.M.); zupko.istvan@szte.hu (I.Z.); 5 MTA-ELTE Lendület Ion Mobility Mass Spectrometry Research Group, Institute of Chemistry, ELTE Eötvös Loránd University, Pázmány Péter sétány 1/A, H-1117 Budapest, Hungary; gitta.schlosser@ttk.elte.hu

**Keywords:** vindoline, imatinib, erlotinib, chalcone, hybrid molecules, Sonogashira reaction, antiproliferative effect, selectivity, ovarian cancer

## Abstract

In the frame of our diversity-oriented research on multitarget small molecule anticancer agents, utilizing convergent synthetic sequences terminated by Sonogashira coupling reactions, a preliminary selection of representative alkyne-tethered vindoline hybrids was synthesized. The novel hybrids with additional pharmacophoric fragments of well-documented anticancer agents, including FDA-approved tyrosine-kinase inhibitors (imatinib and erlotinib) or ferrocene or chalcone units, were evaluated for their antiproliferative activity on malignant cell lines MDA-MB-231 (triple negative breast cancer), A2780 (ovarian cancer), HeLa (human cervical cancer), and SH-SY5Y (neuroblastoma) as well as on human embryonal lung fibroblast cell line MRC-5, which served as a reference non-malignant cell line for the assessment of the therapeutic window of the tested hybrids. The biological assays identified a trimethoxyphenyl-containing chalcone-vindoline hybrid (**36**) as a promising lead compound exhibiting submicromolar activity on A2780 cells with a marked therapeutic window.

## 1. Introduction

**Figure 1:**
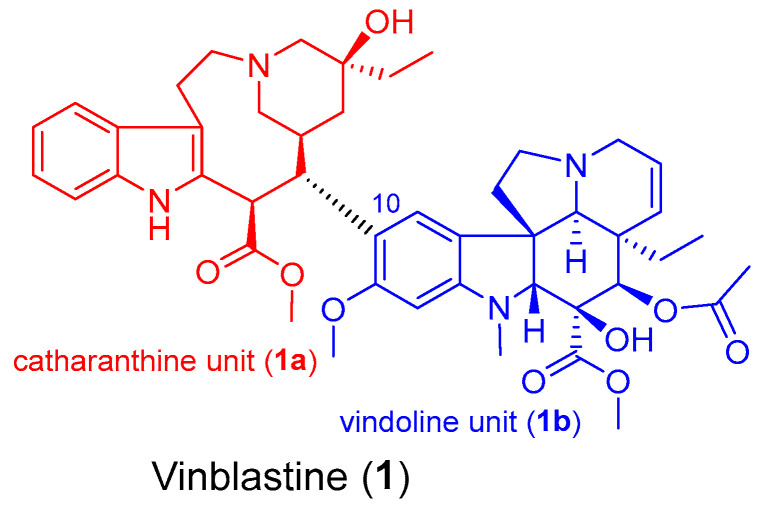
Structure of vinblastine (**1**), composed of the coupled units catharanthine (**1a**) and vindoline (**1b**).

Cancer is one of the most severe health problems. Even though the associated survival rate has improved, different types of tumorous diseases are still among the leading causes of mortality, with low survival rates [1,2]. Although in clinical practice, chemotherapy is generally considered one of the most widely used tools for cancer treatment, often in combination with other therapies, such as surgery, radiation, or hormone therapy, the efficacy of most anticancer chemotherapies is decreased by several therapy-limiting factors, including multidrug resistance (MDR) [3,4] and adverse effects. Consequently, developing more potent novel drugs possessing improved activity, selectivity, and enhanced potency to overcome MDR is continuously the focus of research. One of the most appealing new strategies for developing improved chemotherapy is the fragment-based design and synthesis of hybrid compounds by coupling a reasonable selection of pharmacophoric fragments [5,6,7]. Such hybrid drugs capable of interacting with more than one cellular molecular target can be considered highly potent anticancer agents with enhanced efficiency as they trigger cell death by multiple mechanisms, thus having a real potential to overcome the typical disadvantages of single anticancer agents, including resistance and adverse effects. For an expansion of novel potent therapeutic agents, the implication of compounds of natural origin and their chemically modified versions also seems an attractive strategy. In this regard, several representatives from alkaloid families are of pronounced interest [8,9,10,11]. The representative tubulin inhibitor bis-indole alkaloid Vinblastine (**1**: Figure 1) can also be considered a hybrid composed of the coupled monomers catharanthine (**1a**) and vindoline (**1b**). This alkaloid is one of the most widely used therapeutic agents for treating cancers, e.g., breast cancer [12], acute lymphocytic leukemia [13], and testicular germ-cell carcinomas [14] either as a single agent or in combination with other drugs, exerting its activity by inhibiting tubulin polymerization [15].

**Figure 2:**
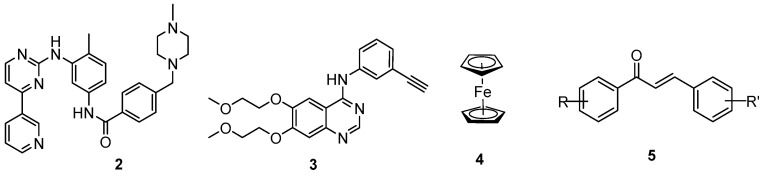
Representative pharmacophores, or their fragments, which were selected for introduction in the targeted vindoline hybrids: FDA-approved kinase inhibitors (imatinib **2** and erlotinib **3**), ferrocene (**4**), and chalcone (**5**).

Vinca alkaloids, including vinblastine, can be isolated from *Catharanthus roseus*. The monomers occur in much greater quantity in the plant than the dimers; however, these single fragments do not display perceptible anticancer effects [16,17]. To address this problem, various vinca alkaloids have been synthesized as hybrids, such as amino acid- and steroid-containing hybrids, and with other pharmacophore moieties [18,19,20]. In this context, pursuing our diversity-oriented research on the development of novel small molecule anticancer agents with enhanced anticancer potency, we aimed at constructing a preliminary selection of novel alkyne-tethered vindoline-based hybrids in which position 10 is coupled to fragments of well-documented pharmacophore residues replacing catharanthine (**1a**), which is present in vinblastine **1**. Accordingly, we planned to introduce the FDA-approved tyrosine-kinase inhibitor anticancer agents imatinib (**2**) [21] and erlotinib (**3**) [22] as well as the ferrocene- and chalcone-containing moieties **4** and **5** (Figure 2) in the targeted vindoline hybrids.

Besides its commercial availability and enhanced stability compared to catharanthine, the choice of vindoline as an essential fragment in our targeted hybrids can be justified by the results published over recent years pointing to the possibility of types of structural engineering that can produce real anticancer agents incorporating this alkaloid conjugated to suitable pharmacophores including amino acids [23], steroids [18], and *N*-heterocycles [19].

The introduction of ferrocene-containing fragments can be justified by the following findings. In 1984, Köpf-Maier et al. reported the anticancer properties of ferrocene salts [24]. Later, more studies reported ferrocene derivatives exhibiting antiproliferative effects on several cancer cell lines and low toxicity against non-transformed cells. It is of importance that due to its stability, super-aromaticity, elevated membrane-penetrating ability, and implication in substituent-dependent, and thus fine-tunable, ROS-generating single electron transfer (SET) events, organoferrocene fragments became the most widespread structural motifs in the emerging group of organometallics displaying anticancer activity triggered by versatile mechanisms of action [25,26,27,28,29,30,31,32,33,34,35]. It has also been demonstrated that replacing the aromatic nucleus of certain organic compounds for a ferrocene unit can lead to products with antiproliferative activity that is absent or less manifested in the parent molecule [36,37,38,39]. 

Chalcones are also privileged scaffolds embedded in a plethora of highly potent anticancer drug candidates inducing cancer cell death by versatile mechanisms of action, including cell cycle arrest in the subG1, S, and G2/M phases, inhibition of tubulin polymerization, enzyme dynamics [40,41,42,43,44,45], and signal transductions initiated by nuclear factor κB [46]. On the other hand, it is also of pronounced importance that various chalcone-containing scaffolds feature marked potency even in overcoming drug resistance, as they were found to exhibit in vitro and in vivo effects on both drug-susceptible and drug-resistant cancers by targeting the aromatase enzyme (CIP19A1), breast cancer resistance protein (BCRP), vascular endothelial growth factor (VEGF), and ATP binding cassette subfamily G member 2 (ABCG2) [47,48]. Since indole is an integrated structural motif in vindoline, chalcone–indol hybrids are particularly worth highlighting as further examples with demonstrated antiproliferative activity against A549, MCF-7, HepG2, paclitaxel-resistant HCT-8/T, and vincristine-resistant HCT-8/V cell lines [49]. In this regard, Yan et al. reported the low nanomolar activity of chalcone–indol hybrids detected, again, with A549, MCF-7, and HCT-8 cancer cells [50].

## 2. Results and Discussion

The targeted alkyne-tethered hybrids were synthesized via two straightforward convergent synthetic pathways both terminated by Sonogashira reactions involving the readily available 10-iodovindoline [51] and the primarily prepared propargylated pharmacophoric moieties, or the 10-iodovindoline-derived silyl-protected 10-ethynylvindoline and iodinated chalcones as alternative coupling partners. Besides their synthetic aspects, the introduction of an acetylenic linker into anticancer drug candidates can also be beneficial in terms of their bioactivity, as justified by characteristic literature examples reporting on alkyne derivatives identified as potent antitumor agents of natural and synthetic origins [52,53,54,55,56,57,58]. A representative alkyne-coupled pyrrolo[2,3-*d*]pyrimidine, BIIB028, displaying therapeutic activity with excellent drug-like properties and an acceptable safety profile in the treatment of breast cancer, melanoma, gastrointestinal cancer, lymphoma, and myeloma [59,60,61], further supports the view about the benefits of adding carbon-carbon triple bonds as rigid spacers into potential anticancer agents.

### 2.1. Multistep Synthesis of the Alkyne-Tethered Vindoline Hybrids

#### 2.1.1. Synthesis of Propargylated Imatinib Fragments

**Scheme 1:**
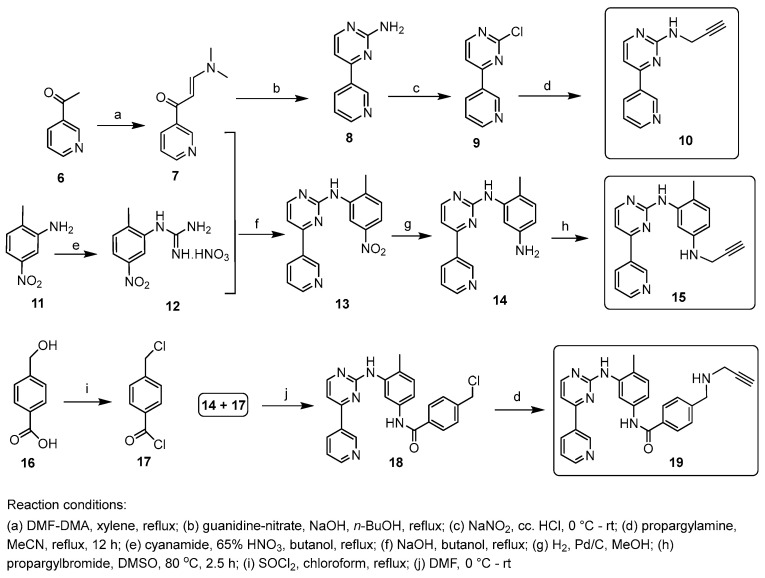
Synthesis of propargylated imatinib fragments suitable for Sonogashira reactions with 10-iodovindoline.

The propargylated imatinib fragments were synthesized utilizing the feasibility of the introduction of propargyl groups into the intermediates **9**, **14,** and **18,** synthesized by the reaction sequences developed by Liu et al. [62], as shown in Scheme 1. Accordingly, 3-acetylpyridine (**6**) was reacted with DMF-DMA to obtain the intermediate enaminone **7,** which was cyclized with guanidine-nitrate to obtain aminopyrimidine **8**, the diazotization followed by chlorination of which formed chloropyrimidine **9**. This electrophilic intermediate was reacted with propargylamine to obtain compound **10**, an alkyne-functionalized imatinib moiety suitable for Sonogashira coupling. Using another pathway, the pyrimidine-forming ring closure of enaminone **7** was performed with *N*-(2-methyl-5-nitrophenyl)guanidine nitrate **12,** previously generated from nitroaniline **11**. The catalytic hydrogenation of the resulting nitroaryl derivative **13** gave aniline **14** as a nucleophilic key intermediate, the *N*-alkylation of which with propargyl bromide conducted under standard conditions led to the formation of the next imatinib-based terminal alkyne **15,** featuring a more extended structural motif compared to the biaryl-type intermediate **10**. Finally, the reaction pathway starting with the double chlorination of 4-(hydroxymethyl)benzoic acid (**16** → **17**) followed by *N*-acylation (**14** + **17** → **18**) and the sequential *N*-propargylation of the benzyl chloride-type intermediate **18** created **19**, the most complex propargylated fragment comprising the majority of the structural motifs of imatinib.

#### 2.1.2. Sonogashira Coupling Reactions Terminating the Synthetic Pathways to the Targeted Alkyne-Tethered Vindoline Hybrids

**Scheme 2:**
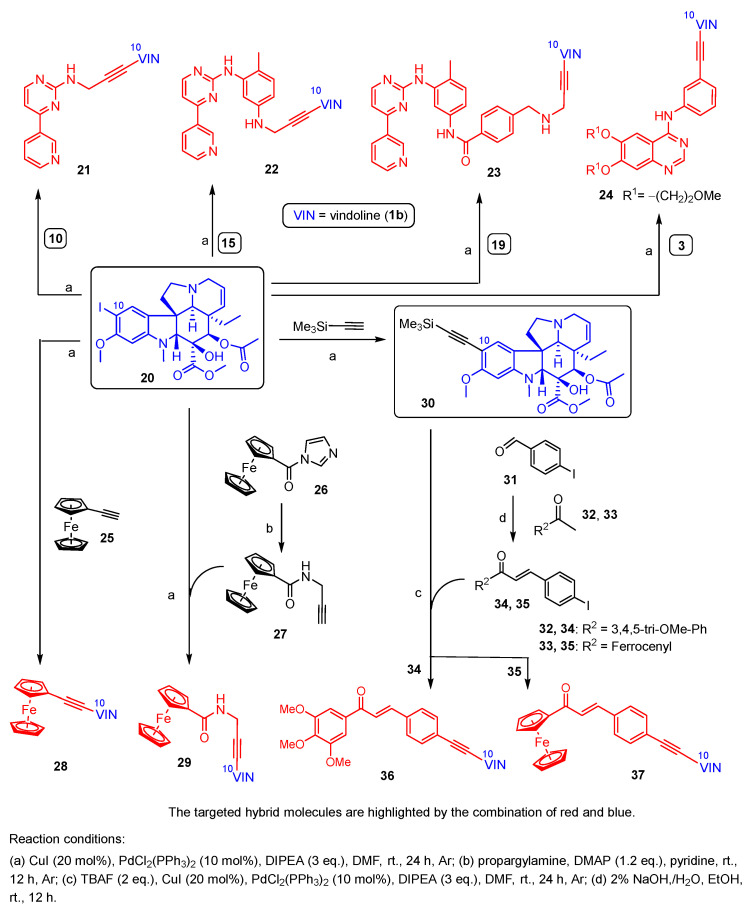
Synthetic pathways to the targeted hybrids starting from 10-iodovindoline (**20**) as common precursor.

The first group of the hybrids containing the fragments of FDA-approved anticancer agents **21**–**24** was synthesized by coupling 10-iodovindoline **20** with propargyalated imatinib fragments (**10**, **15, 19**) and intact erlotinib (**3**), as outlined in Scheme 2. The reactions were conducted for 24 h at room temperature in DMF using CuI(20%)/PdCl_2_(PPh_3_)_2_(10%) and *N*,*N*-diisopropylethylamine (DIPEA) (3 eq.) as a catalyst system and base, respectively (Scheme 2). Under the same conditions, ferrocene-containing hybrids **28** and **29** were obtained when the commercially available ethynylferrocene **25** and *N*-propargyl ferrocene carboxamide **27**, respectively, were used as alkyne components in the coupling reactions. The carboxamide **27** was obtained by the well-established acylation of propargylamine with *N*-ferrocenoylimidazole **26** [63].

In the first step of the synthetic route to creating the representative chalcone-containing hybrids, 10-iodovindoline **20** was coupled with trimethylsilylacetylene under the same Sonogashira reaction conditions to create protected alkyne **30**. In a one-pot procedure, without isolation and purification, the unstable **30** was subjected to TBAF-mediated desilylation followed by Sonogashira coupling of the resulting non-isolated 10-ethynylvindoline with iodinated chalcones **34** and **35** to obtain hybrids **36** and **37**, respectively (Scheme 2). Iodochalcones **34** and **35** were previously prepared by Claisen–Schmidt condensation of 4-iodobenzaldehyde **31** with 3,4,5-trimethoxyacetophenone (**32**) and acetylferrocene (**33**), respectively (Scheme 2).

**Scheme 3:**
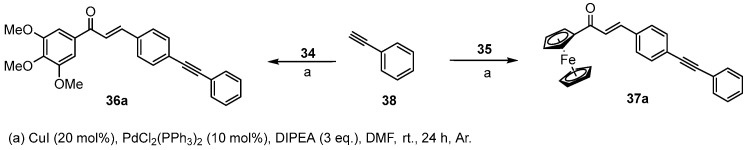
Synthesis of reference chalcones containing phenyl group in place of vindoline moiety.

The reactions of **34** and **35** with phenylacetylene **38** led to the formation of chalcones **36a** and **37a** (Scheme 3), serving as reference models in the biological assays.

### 2.2. In Vitro Antiproliferative Evaluation of the Novel Vindoline Hybrids and Reference Compounds

The antiproliferative effect of the novel vindoline-containing hybrids was initially tested on MRC-5 cells (non-cancerous human embryonal lung fibroblasts) to obtain results concerning the cytotoxicity of the tested molecules. The anticancer properties of the substances were characterized utilizing MDA-MB-231 (breast adenocarcinoma), HeLa (human cervical cancer), A2780 (ovarian cancer), and SH-SY5Y (neuroblastoma) cells. Two concentrations, 10 and 30 µM, were applied at the initial screening. In the case of the most potent analogs, i.e., when the cell growth inhibition was higher than 50% at 10 µM on any cancer cell line, the assay was repeated with a set of dilutions to determine the IC_50_ values. The results of the growth inhibition screening and the IC_50_ values of the most potent compounds are listed in Table 1 and Table 2, respectively.

The percentage of cell growth inhibition caused by vindoline (**1b**) and the newly synthesized hybrids (**21**–**24**, **28**, **29**, **36**, **37**, **36a,** and **37a**) are listed in Table 1. It can be seen that vindoline extended with pharmacophore units shows some cytotoxic effects. Of the hybrids containing imatinib fragments **(21**–**23**), component **22** showed cell division inhibition above 85% with three cell lines (MDA-MB-231, A2780, SH-SY5Y) at a concentration of 30 µM. The ferrocene-containing compounds (**28** and **29**) did not elicit substantial cell division inhibitory effects at a concentration of 30 µM.

Trimethoxyphenyl derivative **36** was identified as the most potent antiproliferative agent (IC_50_ = 0.6–2.55 µM), especially against the A2780 cell line. A comparison of the effects of **36** and its simplified analog **36a** led to the conclusion that the contribution of the vindoline residue to the antiproliferative effect on malignant cells is 3–5-fold more substantial than that of the phenyl group (Table 2). These most promising compounds were comparable to the reference agent cisplatin. Though the IC_50_ values measured with the malignant and MRC-5 cells show comparable therapeutic windows for **36** and **36a**, the latter seems less toxic against non-cancerous cells. However, it must be emphasized that based on the ratio of the IC_50_ values (MRC-5/A2780 = 4.25 and 3.35 for **36** and **36a**, respectively), A2780 ovarian cancer cells are particularly susceptible to these novel agents. The exact identification of the potential therapeutic target requires further investigation, including in vivo experiments; however, at this stage of our research in which we are collecting data for establishing SAR followed by performing mechanistic studies and designing more potent drug candidates, it can only be assumed that hybrid **36** exerts its effect by dual-targeting the vinca and colchicine sites of tubulin heterodimers, as supported by the following facts. In the vinblastine molecule, the vindoline domain was found to bind to the vinca site [65], and the trimethoxyphenyl group is a typical fragment present in a large number of tubuline-targeting anticancer agents, including a variety of chalcones and combretastatin analogs, such as the FDA-approved drug fosbretabulin, all of which bind to the colchicine site [66]. The concept of simultaneously targeting two binding sites of microtubules was also exploited by Passarella et al., who synthesized hybrids comprising vinca alkaloids and other documented polymerization inhibitors [67]. The hybrids containing vindoline tethered at position 17 to thiocolchicine via diacyl spacers of different lengths demonstrated the significant inhibition of tubulin polymerization and antiproliferative activity on A549 lung cancer cells [67]. Finally, our view about the possible molecular targets of **36** is in good accordance with the significantly reduced effect produced by **37** in which the trimethoxyphenyl group is replaced by three-dimensional ferrocene. However, the IC_50_ values produced by **36a** and **37a** indicate that the same structural modification caused a smaller decrease in the effect of phenylacetylene-derived hybrids than in the vindoline-containing analogs. Finally, it must be pointed out that our lead compound **36** proved to be superior to cisplatin with each investigated cell line in terms of both the antiproliferative effect and selectivity, as indicated by the data listed in Table 2.

## 3. Materials and Methods

All chemicals were obtained from commercially available sources (Merck, Budapest, Hungary; Fluorochem, Headfield, UK; Molar Chemicals, Halásztelek, Hungary; VWR, Debrecen, Hungary) and used without further purifications. Equipment from Merck Kieselgel (230–400 mesh, 60 Å) was used for flash column chromatography. Melting points (uncorrected) were determined with a Büchi M-560. The ^1^H- and ^13^C-NMR spectra were recorded in DMSO-*d*_6_ solution in 5 mm tubes at room temperature on a Bruker DRX-500 spectrometer (Bruker Biospin, Karlsruhe, Baden Württemberg, Germany) at 500 (^1^H) and 125 (^13^C) MHz, with the deuterium signal of the solvent as the lock and TMS as internal standard (^1^H and ^13^C). The 2D-HSQC-, HMBC-, and NOESY spectra, which support the exact assignments of ^1^H- and ^13^C NMR signals, were measured by using the standard Bruker pulse programs. Exact mass measurements were performed on a high-resolution Waters ACQUITY RDa Detector (Waters Corp., Wilmslow, UK) equipped with an electrospray ionization source using on-line UHPLC coupling. UHPLC separation was performed on a Waters ACQUITY UPLC H-Class PLUS system using a Waters Acquity UPLC BEH C18 column (2.1 × 150 mm, 1.7 µm). Samples were dissolved in MeOH/Water 5:95 *v/v*, and 5-5 µL sample solutions were injected. Linear gradient elution (0 min 5% B, 1.0 min 5% B, 7.0 min 80% B, 7.1 min 100% B, 8.0 min 100% B, 8.1 min 5% B, 12.0 min 5% B) with eluent A (0.1% formic acid in water, *v/v*) and eluent B (0.1% formic acid in Methanol, *v/v*) was used at a flow rate of 0.200 mL/min at 45 °C column temperature. High-resolution mass spectra were acquired in the *m*/*z* 50–2000 range in the positive ionization mode. Leucine enkephalin peptide was used for single lock mass calibration correction.

For each compound characterized in this study, the numbering of atoms used for the assignment of ^1^H- and ^13^C-NMR signals do not correspond to the IUPAC rules reflected in the given systematic names. Imatinib fragments (**7–9, 12–14, 17,** and **18**) were synthesized by reported procedures [62]. *N*-Ferrocenoylimidazole (**26**) was prepared using the method reported by Imrie et al. [63].

Copies of the NMR spectra of the novel propargylated pharmacophore fragments (S2–S5), the novel iodinated chalcones (S6–S7) and the novel alkyne-tethered vindoline hybrids (S8–S16) along with the copies of the HRMS spectra of the novel alkyne-tethered vindoline hybrids (S17–S20) are included in the Supplementary Materials.

### 3.1. General Procedure for the Synthesis of Propargylated Imatinib Fragments ***10*** and ***19***

2-Chloro-4-(pyridine-3-yl)pyrimidine (**9**) or 4-(chloromethyl)-*N*-(4-methyl-3-((4-(pyridine-3-yl)pyrimidine-2-yl)amino)phenyl)benzamide (**18**) (2 mmol) was dissolved in MeCN (20 mL), then propargylamine (1.27 mL, 1.10 g, 20 mmol) was added dropwise to the solution. The obtained mixture was stirred at reflux temperature for 12 h and concentrated in vacuo. The residue was purified by column chromatography on silica using solvent mixture DCM:MeOH (15:1) as eluent, followed by crystallization with Et_2_O to obtain the pure product (**10**, **19**).

#### 3.1.1. N-(Prop-2-yn-1-yl)-4-(pyridin-3-yl)pyrimidin-2-amine (**10**)


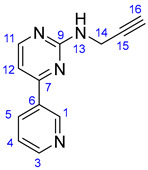


Light grey solid. Mp: 93–95 °C; yield: 104 mg (26%). ^1^H-NMR (DMSO-*d*_6_): 9.30 (br s, 1H, H1); 8.65 (dd, *J* = 4.5 Hz and 1.6 Hz, 1H, H3); 8.46 (two coalesced d’s, *J* = 5.0 Hz, 2H, H5 and H11); 7.67 (t, *J* = 5.9 Hz, 1H, H13); 7.55 (dd, *J* = 7.8 Hz and 4.6 Hz, 1H, H4); 7.32 (d, *J* = 5.1 Hz, H12); 4.15 (dd, *J* = 5.9 Hz and 2.2 Hz, 1H, 2H, H14); 3.02 (t, *J* = 2.2 Hz, 1H, H17). ^13^C-NMR (DMSO-*d*_6_): 164.9 (C7); 162.3 (C9); 159.9 (C11); 151.9 (C3); 148.6 (C1); 134.8 (C5); 133.0 (C6); 124.3 (C4); 107.2 (C12); 82.7 (C15); 72.6 (C17); 30.8 (C14);

#### 3.1.2. N-(4-Methyl-3-((4-(pyridin-3-yl)pyrimidin-2-yl)amino)phenyl)-4-((prop-2-yn-1-ylamino)-methyl)-benzamide (**19**)


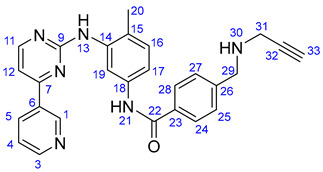


Light orange solid. Mp: 155–156 °C; yield: 843 mg (94%). ^1^H-NMR (DMSO-*d*_6_): 10.17 (s, 1H, H21); 9.22 (d, *J* = 1.6 Hz, 1H, H1); 8.91 (s, 1H, H13); 8.65 (dd, *J* = 4.6 Hz and 1.6 Hz, 1H, H3); 8.48 (d, *J* = 5.1 Hz, 1H, H11); 8.44 (dt, *J* = 7.8 Hz and 1.6 Hz, 1H, H5); 7.92 (*d*, *J* = 8.5 Hz, 2H, H24 and H28); 7.50–7.46 (overlapping m’s, 4H, H4, H17, H25 and H27); 7.33 (d, *J* = 5.1 Hz, 1H, H12); 6.93 (d, *J* = 8.2 Hz, 1H, H16); 6.86 (d, *J* = 2.0 Hz, 1H, H19); 3.92 (s, 2H, H29); 3.44 (d, *J* = 2.0 Hz, 2H, H31); 3.24 (t, *J* = 2.0 Hz, 1H, H33); 2.04 (s, 3H, H20); ^13^C-NMR (DMSO-*d*_6_): 165.7 C22); 164.9 (C7); 162.3 (C7); 161.6 (C9); 159.2 (C11); 151.9 (C3); 148.6 (C1); 141.6 (C26); 138.3 (C14); 137.7 (C18); 135.1 (C5); 132.5 (C6); 130.8 (C16); 129.4 (C23); 129.0 (C25 and C27); 128.1 (C24 and C28); 126.4 (C15); 124.4 (C4); 117.9 (C19); 117.5 (C17); 107.8 (C12); 81.0 (C32); 76.0 (C33); 50.8 (C29); 36.9 (C31); 18.1 (C20).

### 3.2. Synthesis of 4-Methyl-N^1^-(prop-2-yn-1-yl)-N^3^-(4-(pyridine-3-yl)pyrimidine-2-yl)benzene-1,3-diamine (***15***)

6-Methyl-*N*-(4-(pyridine-3-yl)pyrimidine-2-yl)benzene-1,3-diamine (**14**) (1.109 g, 4 mmol) and NaHCO_3_ (336 mg, 4 mmol) were dissolved in DMSO (12 mL). To this solution, propargylbromide (0.37 mL, 571 mg, 4.8 mmol) was added dropwise, and the reaction mixture was stirred for 2.5 h at 70 °C. The mixture was poured into water and extracted with DCM (3 × 25 mL). The combined organic phase was washed with brine and water, then dried over Na_2_SO_4_ and evaporated to dryness. The residue was subjected to column chromatography on silica using EtOAc:MeOH:TEA (10:1:0.03) as eluent, then crystallized from Et_2_O to obtain the pure product as a yellow powder.


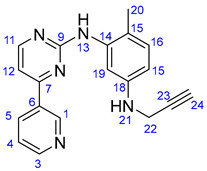


Light grey solid. Mp: 101–103 °C (dec.); yield: 215 mg (17%). ^1^H-NMR (DMSO-*d*_6_): 9.22 (d, *J* = 1.6 Hz, 1H, H1); 8.68 (s, 1H, H13); 8.65 (dd, *J* = 4.6 Hz and 1.6 Hz, 1H, H3); 8.43 (d, *J* = 5.1 Hz, 1H, H11); 8.34 (dt, *J* = 7.8 Hz and 1.6 Hz, 1H, H5); 7.48 (dd, *J* = 7.8 Hz and 4.6 Hz, 1H, H4); 7.33 (*d*, *J* = 5.1 Hz, 1H, H12); 6.94 (d, *J* = 8.3 Hz, 1H, H16); 6.86 (d, *J* = 2.0 Hz, 1H, H19); 6.38 (dd, *J* = 8.3 Hz and 2.0 Hz, 1H, H15); 5.73(t, *J* = 6.1 Hz, 1H, H21); 3.78 (dd, *J* = 6.1 Hz and 2.1 Hz, 2H, H22); 2.96 (t, *J* = 2.1 Hz, 1H, H24); 2.05 (s, 3H, H20); ^13^C-NMR (DMSO-*d*_6_): 162.0 (C7); 161.2 (C9); 160.0 (C11); 151.9 (C3); 148.7 (C1); 146.7 (C18); 138.7 (C14); 134.8 (C5); 132.7 (C6); 130.8 (C16); 124.3 (C4); 120.9 (C15); 110.4 (C19); 110.0 (C15); 107.8 (C12); 83.1 (C23); 73.3 (C24); 33.0 (C22); 17.7 (C20).

### 3.3. Synthesis of N-Propargylferrocenecarboxamide (***27***)

Propargylamine (0.32 mL, 0.275 g, 5 mmol), ferrocenoylimidazolide **26** (1.704 g; 6 mmol, 1.2 eq.), and DMAP (0.184 g; 6 mmol, 1.2 eq.) were dissolved in freshly distilled pyridine (15 mL). This reaction mixture was purged with argon and stirred for 12 h at room temperature, then poured onto crushed ice. The resulting suspension was extracted with DCM (5 × 20 mL). The combined organic layers were washed with brine solution, dried over anhydrous Na_2_SO_4_, and evaporated to dryness on a rotary evaporator. The dark solid residue was purified by column chromatography on silica using solvent mixture DCM:MeOH (20:1) as eluent, followed by sequential crystallization with water and Et_2_O to obtain the pure product as a light orange solid. Yield: 1.00 g (75%).


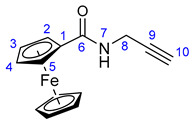


Light orange powder. Mp: 125–126 °C (dec.); ^1^H-NMR (DMSO-*d*_6_): 8.30 (t, *J* = 5.6 Hz, 1H, H7); 4.83 (t, *J* = 1.8 Hz, 2H, H2 and H5); 4.35 (t, *J* = 1.8 Hz, 2H, H3 and H4); 4.18 (s, 5H, η^5^-C_5_H_5_); 3.95 (dd, *J* = 5.6 Hz and 2.4 Hz, 2H, H8); 3.11 (t, *J* = 2.4 Hz, 1H, H10). ^13^C-NMR (DMSO-*d*_6_): 169.2 (C6); 82.7 (C9); 76.2 (C1); 69.8 (η^5^-C_5_H_5_); 69.6 (C4 and C5); 64.7 (C2 and C5); 28.4 (C8).

### 3.4. Synthesis of Iodochalcone Intermediates ***34*** and ***35***

The corresponding methyl ketone **32** or **33** (1 mmol) and 4-iodobenzaldehyde **31** (232 mg, 1 mmol) were dissolved in EtOH (3 mL). To this solution, 2% NaOH/H_2_O (2 mL) was added, and the resulting mixture was stirred for 12 h at room temperature under argon atmosphere. The precipitated crystals were filtered out and first purified by column chromatography on silica using solvent mixture DCM:MeOH (40:1) as eluent and crystallized from EtOH to obtain the pure product.

#### 3.4.1. (E)-3-(4-Iodophenyl)-1-(3,4,5-trimethoxyphenyl)prop-2-en-1-one (**34**)


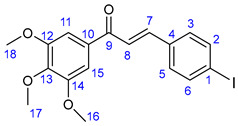


Pale yellow crystals. Mp: 182–184 °C (dec.); yield: 390 mg (87%). Yield: 391 mg (87%). ^1^H-NMR (DMSO-*d*_6_): 7.98 (d, *J* = 15.6 Hz, 1H, H8); 7.84 (d, *J* = 8.2 Hz, 2H, H2 and H6); 7.71 (d, *J* = 8.2 Hz, 2H, H3 and H5); 7.68 (d, *J* = 15.6 Hz, 1H, H7); 7.43 (s, 2H, H11 and H15); 3.90 (s, 6H, H16 and H18); 3.77 (s, 3H, H17). ^13^C-NMR (DMSO-*d*_6_): 188.2 (C9); 153.4 (C12 and C14); 143.2 (C7); 142.6 (C13); 138.2 (C2 and C6); 134.7 (C4); 133.3 (C10); 131.3 (C3 and C5); 123.3 (C8); 106.7 (C11 and C15); 98.1 (C1); 60.7 (C17), 56.6 (C16 and C18).

#### 3.4.2. (E)-3-(4-Iodophenyl)-1-ferrocenylprop-2-en-1-one (**35**)


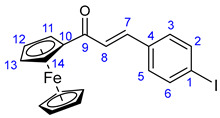


Deep red crystals. Mp: 133–135 °C (dec.); yield: 314 mg (71%). ^1^H-NMR (DMSO-*d*_6_): 7.82 (d, *J* = 8.0 Hz, 2H, H2 and H6); 7.67 (d, *J* = 8.0 Hz, 2H, H3 and H5); 7.56 (d, *J* = 15.6 Hz, 1H, H7); 7.46 (d, *J* = 15.6 Hz, 1H, H8); 5.04 (br s, 2H, H11 and H14); 4.67 (br s, 2H, H12 and H13); 4.21 (s, 5H, η^5^-C_5_H_5_). ^13^C-NMR (DMSO-*d*_6_): 192.4 (C9); 139.1 (C7); 138.2 (C2 and C6); 135.0 (C4); 131.0 (C3 and C5); 124.8 (C8); 97.1 (C1); 81.1 (C10); 73.3 (C12 and C13); 70.2 (C11 and C14); 70.3 (η^5^-C_5_H_5_).

### 3.5. General Procedure for the Sonogashira Reactions Using 10-Iodovindoline (***20***) as Coupling Partner and Synthesis of Hybrids ***21***–***24***, ***28***, ***29*** and Silyl-Protected Intermediate ***30***

10-Iodovindoline (**20**) (1 mmol), the corresponding alkyne component (**3**, **10**, **15**, **19**, **25**, **27** or trimethylsilylacetylene) (1 mmol), CuI (38 mg, 0.2 mmol), PdCl_2_(PPh_3_)_2_ (70 mg, 0.1 mmol), and DIPEA (0.53 mL, 390 mg, 3 mmol) were dissolved in DMF (20 mL). The mixture was stirred for 24 h at room temperature under argon atmosphere then poured into water. The precipitate was filtered off, washed with water (5 × 10 mL), dried, extracted with DCM (4 × 15 mL), and filtered off again. The organic solution was washed with water (1 × 30 mL) and brine, then dried over Na_2_SO_4_. After evaporation of the solvent, the solid residue was subjected to column chromatography on silica using solvent mixture DCM:MeOH (15:1) as eluent and crystallized from Et_2_O to obtain the pure product. Since over the course of chromatography, **30** underwent decomposition, this silylated intermediate was used without purification for subsequent Sonogashira reactions with the iodochalcones.

#### 3.5.1. Methyl (3aR,3a^1^R,4R,5S,5aR,10bR)-4-Acetoxy-3a-ethyl-5-hydroxy-8-methoxy-6-methyl-9-(3-((4-methyl-3-((4-(pyridin-3-yl)pyrimidin-2-yl)amino)phenyl)amino)prop-1-yn-1-yl)-3a,3a^1^,4,5,5a,6,11,12-octahydro-1H-indolizino[8,1-cd]carbazole-5-carboxylate (**21**)


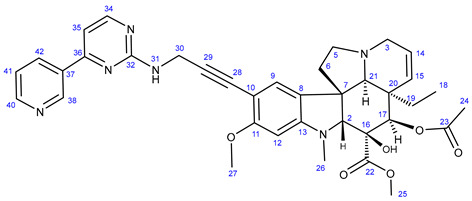


Light yellowish solid. Mp: 162–163 °C; yield: 160 mg (24%). ^1^H-NMR (DMSO-*d*_6_): 9.10 (d, *J* = 2.0 Hz, 1H, H38); 8.80 (s, 1H, OH on C16); 8.65 (dd, *J* = 4.6 Hz and 1.6 Hz, 1H, H40); 8.58 (d, *J* = 5.1 Hz, 1H, H42); 8.49 (d, *J* = 5.3 Hz, 1H, H34); 7.52 (dd, *J* = 7.8 Hz and 4.6 Hz, 1H, H41); 7.21 (s, 1H, H9); 7.13 (d, *J* = 5.3 Hz, 1H, H35); 6.26 (s, 1H, H12); 6.24 (t, *J* = 5.0 Hz, 1H, H31); 5.83 (ddd, *J* = 9.9 Hz, 4.8 Hz and 1.8 Hz, 1H, H14); 5.14 (s, 1H, H17); 5.11 (d, *J* = 9.9 Hz, 1H, H15); 4.03 (d, *J* = 5.0 Hz, 1H, H30); 3.77 (s, 3H, H27); 3.62 (s, 3H, H25); 3.58 (s, 1H, H2); 3.42 (m, 1H, H3/A); 3.28 (m, 1H, H5/A); 2.91 (br d, *J* = 16.2 Hz, 1H, H3/B); 2.72, (s, 1H, H21); 2.68 (m, 1H, H5/B); 2.59 (s, 3H, H26); 2.24 (m, 2H, H6A and H6B); 1.92 (s, 3H, H24); 1.49 (m, 1H, H19/A), 0.95 (m, 1H; H19/B); 0.46 (t, *J* = 6.9 Hz, 3H, H18). ^13^C-NMR (DMSO-*d*_6_): 172.6 (C22); 170.7 (C23); 163.1 (C36); 161.4 (C11); 157.2 (C34); 156.5 (C32); 153.4 (C13); 151.7 (C40); 131.2 (C37); 130.3 (C15); 127.3 (C9); 125.6 (C8); 124.8 (C14); 124.0 (C41); 103.9 (C35); 101.7 (C10); 93.4 (C12); 91.2 (C29); 80.8 (C2); 81.3 (C28); 76.3 (C17); 65.5 (C21); 60.4 (C5); 58.4 (C27); 53.0 (C7); 51.5 (C25); 50.5 (C3); 43.6 (C6); 42.5 (C20); 39.2 (C26); 30.4 (C19); 21.1 (C24); 8.1 (C18).

#### 3.5.2. Methyl (3aR,3a^1^R,4R,5S,5aR,10bR)-4-Acetoxy-3a-ethyl-5-hydroxy-8-methoxy-6-methyl-9-(3-((4-methyl-3-((4-(pyridin-3-yl)pyrimidin-2-yl)amino)phenyl)amino)prop-1-yn-1-yl)-3a,3a^1^,4,5,5a,6,11,12-octahydro-1H-indolizino[8,1-cd]carbazole-5-carboxylate (**22**)


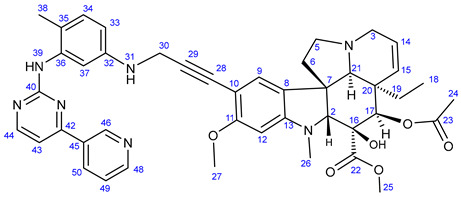


Light yellowish solid. Mp: 157–158 °C; yield:193 mg (25%). ^1^H-NMR (DMSO-*d*_6_): 9.22 (d, *J* = 1.8 Hz, 1H, H55); 8.73 (s, 1H, OH on C16); 8.69 (s, 1H, H39); 8.65 (dd, *J* = 4.6 Hz and 1.6 Hz, 1H, H48); 8.65 (dd, *J* = 4.6 Hz and 1.8 Hz, 1H, H57); 8.43 (d, *J* = 5.1 Hz, 1H, H44); 8.34 (dt, *J* = 7.8 Hz and 1.8 Hz, 1H, H50); 7.48 (dd, *J* = 7.8 Hz and 4.8 Hz, 1H, H49); 7.33 (d, *J* = 5.1 Hz, 1H, H43); 7.12 (s, 1H, H9); 6.93 (d, *J* = 8.2 Hz, 1H, H34); 6.86 (d, *J* = 2.0 Hz, 1H, H37); 6.38 (dd, *J* = 8.2 Hz and 2.0 Hz, 1H, H33); 6.26 (s, 1H, H12); 5.84 (ddd, *J* = 9.9 Hz, 4.8 Hz and 1.8 Hz, 1H, H14); 5.82 (t, *J* = 6.1 Hz, 1H, H31); 5.13 (s, 1H, H17); 5.11 (d, *J* = 9.9 Hz, 1H, H15); 4.05 (d, *J* = 6.1 Hz, 1H, H30); 3.76 (s, 3H, H27); 3.64 (s, 3H, H25); 3.57 (s, 1H, H2); 3.44 (m, 1H, H3/A); 3.31 (m, 1H, H5/A); 2.93 (br d, *J* = 16.2 Hz, 1H, H3/B); 2.72, (s, 1H, H21); 2.63 (m, 1H, H5/B); 2.59 (s, 3H, H26); 2.26 (m, 2H, H6A and H6B); 2.05 (s, 3H, H38); 1.92 (s, 3H, H24); 1.52 (m, 1H, H19/A), 0.95 (m, 1H; H19/B); 0.46 (t, *J* = 6.9 Hz, 3H, H18). ^13^C-NMR (DMSO-*d*_6_): 172.5 (C22); 170.6 (C23); 162.0 (C42); 161.2 (C40); 161.5 (C11); 160.0 (C44); 153.4 (C13); 151.9 (C48); 148.7 (two coalesced lines, C32 and C46); 138.7 (C36); 134.8 (C50); 132.7 (C45); 130.8 (C34); 130.3 (C15); 125.3 (C8); 124.3 (C49); 124.8 (C14); 159.2 (C53); 151.9 (C57); 141.6 (C33); 138.3 (C45); 137.7 (C41); 135.1 (C59); 132.5 (C54); 130.8 (C43); 129.4 (C36); 129.0 (C34 and C38); 128.1 (C35 and C37); 127.4 (C9); 126.4 (C44); 124.4 (two coalesced lines, C8 and C58); 117.9 (C46); 117.5 (C42); 107.8 (C52); 101.7 (C10); 93.2 (C12); 89.9 (C29); 83.5 (C2); 81.3 (C28); 76.3 (C17); 65.5 (C21); 60.4 (C5); 58.4 (C27); 53.0 (C7); 51.5 (two coalesced lines, C25 and C32); 50.5 (C3); 43.6 (C6); 42.5 (C20); 39.2 (C26); 38.2 (C30); 30.4 (C19); 21.1 (C24); 18.3 (C47); 8.1 (C18). Elemental composition: C_44_H_47_N_7_O_6_, HRMS: calculated *m*/*z*: 770.3661, measured *m*/*z*: 770.36549, mass error: 0.8 ppm (M+H^+^).

#### 3.5.3. Methyl (3aR,3a^1^R,4R,5S,5aR,10bR)-4-Acetoxy-3a-ethyl-5-hydroxy-8-methoxy-6-methyl-9-(3-((4-((4-methyl-3-((4-(pyridin-3-yl)pyrimidin-2-yl)amino)phenyl)carbamoyl)benzyl)amino)prop-1-yn-1-yl)-3a,3a^1^,4,5,5a,6,11,12-octahydro-1H-indolizino[8,1-cd]carbazole-5-carboxylate (**23**)


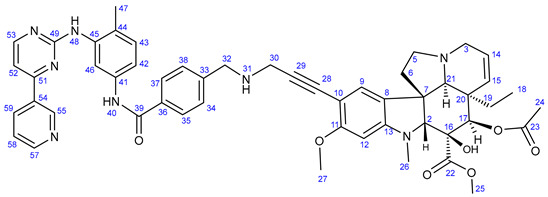


Light orange solid. Mp: 138–140 °C; yield: 181 mg (20%) ^1^H-NMR (DMSO-*d*_6_):10.17 (s, 1H, H40); 9.20 (d, *J* = 1.8 Hz, 1H, H55); 8.90 (s, 1H, H48); 8.74 (s, 1H, OH on C16); 8.65 (dd, *J* = 4.6 Hz and 1.8 Hz, 1H, H57); 8.48 (d, *J* = 5.1 Hz, 1H, H53); 8.44 (dt, *J* = 7.8 Hz and 1.8 Hz, 1H, H59); 8.02 (d, *J* = 2.0 Hz, 1H, H46); 7.94 (d, *J* = 8.5 Hz, 2H, H35 and H37); 7.52 (dd, *J* = 7.8 Hz and 4.8 Hz, 1H, H58); 7.49 (d, *J* = 8.5 Hz, 2H, H34 and H38); 7.44 (dd, *J* = 8.2 Hz and 2.0 Hz, 1H, H42); 7.33 (d, *J* = 5.1 Hz, 1H, H52); 7.17 (s, 1H, H9); 6.93 (d, *J* = 8.2 Hz, 1H, H43); 6.26 (s, 1H, H12); 5.82 (ddd, *J* = 9.9 Hz, 4.8 Hz and 1.8 Hz, 1H, H14); 5.15 (s, 1H, H17); 5.12 (d, *J* = 9.9 Hz, 1H, H15); 3.89 (s, 2H, H32); 3.51 (s, 2H, H30); 3.76 (s, 3H, H27); 3.62 (s, 3H, H25); 3.57 (s, 1H, H2); 3.42 (m, 1H, H3/A); 3.32 (m, 1H, H5/A); 2.91 (br d, *J* = 16.2 Hz, 1H, H3/B); 2.72, (s, 1H, H21); 2.63 (m, 1H, H5/B); 2.57 (s, 3H, H26); 2.26 (m, 2H, H6A and H6B); 2.05 (s, 3H, H47); 1.92 (s, 3H, H24); 1.49 (m, 1H, H19/A), 0.95 m, 1H; H19/B); 0.46 (t, *J* = 6.9 Hz, 3H, H18). ^13^C-NMR (DMSO-*d*_6_): 172.5 (C22); 170.6 (C23); 165.7 (C39); 162.3 (C51); 161.6 (C49); 161.5 (C11); 159.2 (C53); 153.4 (C13); 151.9 (C57); 148.6 (C55); 141.6 (C33); 138.3 (C45); 137.7 (C41); 135.1 (C59); 132.5 (C54); 130.8 (C43); 130.3 (C15); 129.4 (C36); 129.0 (C34 and C38); 128.1 (C35 and C37); 127.4 (C9); 126.4 (C44); 125.4 (C8); 124.8 (C14); 124.4 (two coalesced lines, C8 and C58); 117.9 (C46); 117.5 (C42); 107.8 (C52); 101.7 (C10); 93.2 (C12); 89.9 (C29); 83.5 (C2); 81.3 (C28); 76.3 (C17); 65.5 (C21); 60.4 (C5); 58.4 (C27); 53.0 (C7); 51.5 (two coalesced lines, C25 and C32); 50.5 (C3); 43.6 (C6); 42.5 (C20); 39.2 (C26); 38.2 (C30); 30.4 (C19); 21.1 (C24); 18.3 (C47); 8.1 (C18). Elemental composition: C_52_H_54_N_8_O_7_, HRMS: calculated *m/z*: 903.4188, measured *m/z*: 903.41853, mass error: 0.3 ppm (M+H^+^).

#### 3.5.4. Methyl (3aR,3a^1^R,4R,5S,5aR,10bR)-4-Acetoxy-9-((3-((6,7-bis(2-methoxyethoxy)quinazolin-4-yl)-amino)phenyl)ethynyl)-3a-ethyl-5-hydroxy-8-methoxy-6-methyl-3a,3a^1^,4,5,5a,6,11,12-octahydro-1H-indolizino[8,1-cd]carbazole-5-carboxylate (**24**)


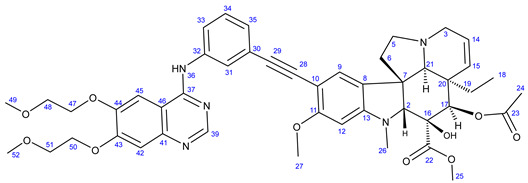


Light orange solid. Mp: 158–159 °C (dec.); yield: 228 mg (27%) ^1^H-NMR (DMSO-*d*_6_): 9.46 (s, 1H, H36); 8.73 (s, 1H, OH on C16); 8.52 (s, 1H, H39); 8.23 (t, *J* = 5.8 Hz, 1H, H31); 7.93 (t, *J* = 2.0 Hz, 1H, H31); 7.87 (dt, *J* = 7.6 Hz and 2.0 Hz, 1H, H35); 7.40 (t, *J* = 7.6 Hz, 1H, H34); 7.23 (s, 2H, H42 and H45); 7.17 (s, 1H, H9); 7.13 (dt, *J* = 7.6 Hz and 2.0 Hz, 1H, H33); 6.26 (s, 1H, H12); 5.82 (ddd, *J* = 9.9 Hz, 4.8 Hz and 1.8 Hz, 1H, H14); 5.15 (s, 1H, H17); 5.12 (d, *J* = 9.9 Hz, 1H, H15); 4.26 (m, 4H, H47 and H50); 3.78 (m, 2H, H48); 3.76 (s, 3H, H27); 3.71 (m, 2H, H51); 3.62 (s, 3H, H25); 3.57 (s, 1H, H2); 3.42 (m, 1H, H3/A); 3.34 (s, 6H, H49 and H52); 3.32 (m, 1H, H5/A); 2.91 (br d, *J* = 16.2 Hz, 1H, H3/B); 2.72, (s, 1H, H21); 2.63 (m, 1H, H5/B); 2.57 (s, 3H, H26); 2.26 (m, 2H, H6A and H6B); 1.92 (s, 3H, H24); 1.49 (m, 1H, H19/A), 0.95 m, 1H; H19/B); 0.46 (t, *J* = 6.9 Hz, 3H, H18). ^13^C-NMR (DMSO-*d*_6_): 172.5 (C22); 170.8 (C23); 161.5 (C11); 156.7 (C37); 154.3 (C43); 153.4 (two coalesced lines, C13 and C39); 148.8 (C44); 147.5 (C41); 140.2 (C32); 130.3 (C15); 129.3 (C34); 127.4 (C9); 126.0 (C8); 125.0 (C33); 124.8 (C14); 124.2 (C31); 124.1 (C30); 109.5 (C46); 108.8 (C42); 104.0 (C45); 101.7 (C10); 93.4 (C12); 91.3 (C29); 88.4 (C28); 83.5 (C2); 79.4 (C16); 76.1 (C17); 70.6 and 70.5 (C47 and C50, interchangeable assignments); 68.9 and 68.6 (C48 and C51, interchangeable assignments); 65.5 (C21); 60.4 (C5); 58.9 (two coalesced lines, C49 and C52); 58.4 (C27); 53.0 (C7); 51.5 (C25); 50.5 (C3); 43.6 (C6); 42.5 (C20); 39.2 (C26); 30.4 (C19); 21.1 (C24); 8.1 (C18). Elemental composition: C_47_H_53_N_5_O_10_, HRMS: calculated *m*/*z*: 848.3865, measured *m*/*z*: 848.38561, mass error: 1.1 ppm (M+H^+^).

#### 3.5.5. Methyl (3aR,3a^1^R,4R,5S,5aR,10bR)-4-Acetoxy-3a-ethyl-5-hydroxy-8-methoxy-6-methyl-9-(ferro-cenylethynyl)-3a,3a^1^,4,5,5a,6,11,12-octahydro-1H-indolizino[8,1-cd]carbazole-5-carboxylate (**28**)


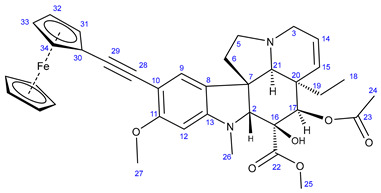


Light orange solid. Mp: 218–219 °C (dec.); yield: 160 mg (24%) ^1^H-NMR (DMSO-*d*_6_): 8.73 (s, 1H, OH on C16); 7.20 (s, 1H, H9); 6.29 (s, 1H, H12); 5.84 (ddd, *J* = 9.9 Hz, 4.8 Hz and 2.0 Hz, 1H, H14); 5.14 (d, *J* = 9.9 Hz, 1H, H15); 5.13 (s, 1H, H17); 4.42 (br s, 2H, H31 and H34); 4.24 (br s, 2H, H32 and H34); 4.20 (s, 5H, η^5^-C_5_H_5_); 3.76 (s, 3H, H27); 3.64 (s, 3H, H25); 3.57 (s, 1H, H2); 3.40 (m, 1H, H3/A); 3.28 (m, 1H, H5/A); 2.91 (br d, *J* = 16.2 Hz, 1H, H3/B); 2.69, (s, 1H, H21); 2.60 (m, 1H, H5B); 2.59 (s, 3H, H26); 2.28 (m, 2H, H6/A and H6/B); 1.92 (s, 3H, H24); 1.49 (m, 1H, H19/A), 0.95 (m, 1H; H19/B); 0.46 (t, *J* = 6.9 Hz, 3H, H18). ^13^C-NMR (DMSO-*d*_6_): 172.5 (C22); 170.6 (C23); 160.2 (C11); 152.9 (C13); 130.3 (C15); 127.4 (C9); 124.8 (C14); 124.4 (C8); 100.1 (C10); 93.4 (C12); 92.1 (C29); 83.0 (C2); 79.1 (C16); 76.3 (C17); 76.2 (C28); 71.5 (C31 and C34); 70.2 (η^5^-C_5_H_5_); 68.9 (C32 and C34); 65.6 (C21); 65.4 (C30); 60.1 (C5); 58.4 (C27); 52.9 (C7); 51.4 (C25); 50.5 (C3); 43.4 (C6); 42.5 (C20); 39.0 (C26); 30.4 (C19); 21.1 (C24); 8.1 (C18); Elemental composition: C_37_H_40_FeN_2_O_6,_ HRMS: calculated *m*/*z*: 664.2230, measured *m*/*z*: 664.22229, mass error: 1.1 ppm (M^+.^, Fe(II) oxidized to Fe(III)).

#### 3.5.6. Methyl (3aR,3a^1^R,4R,5S,5aR,10bR)-4-Acetoxy-9-(3-ferroceneamidoprop-1-yn-1-yl)-3a-ethyl-5-hydroxy-8-methoxy-6-methyl-3a,3a^1^,4,5,5a,6,11,12-octahydro-1H-indolizino[8,1-cd]carbazole-5-carboxylate (**29**)


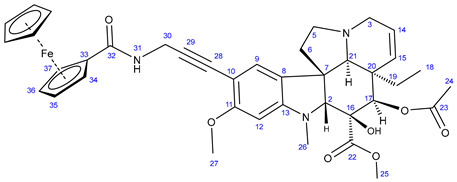


Light orange solid. Mp: 202–204 °C (dec.); yield: 195 mg (27%) ^1^H-NMR (DMSO-*d*_6_): 8.73 (s, 1H, OH on C16); 8.23 (t, *J* = 5.8 Hz, 1H, H31); 7.12 (s, 1H, H9); 6.28 (s, 1H, H12); 5.83 (ddd, *J* = 9.9 Hz, 4.8 Hz and 1.8 Hz, 1H, H14); 5.13 (s, 1H, H17); 5.11 (d, *J* = 9.9 Hz, 1H, H15); 4.52 (br s, 2H, H34 and H37); 4.26 (br s, 2H, H35 and H36); 4.16 (d, *J* = 5.8 Hz, 2H, H30); 4.01 (s, 5H, η^5^-C_5_H_5_); 3.76 (s, 3H, H27); 3.64 (s, 3H, H25); 3.57 (s, 1H, H2); 3.42 (m, 1H, H3/A); 3.30 (m, 1H, H5/A); 2.91 (br d, *J* = 16.2 Hz, 1H, H3/B); 2.68, (s, 1H, H21); 2.60 (m, 1H, H5/B); 2.26 (m, 2H, H6A and H6B); 2.59 (s, 3H, H26); 1.92 (s, 3H, H24); 1.50 (m, 1H, H19/A), 0.96 m, 1H; H19/B); 0.47 (t, *J* = 6.9 Hz, 3H, H18). ^13^C-NMR (DMSO-*d*_6_): 172.5 (C22); 170.6 (C23); 169.3 (C32); 161.8 (C11); 153.7 (C13); 130.3 (C15); 127.4 (C9); 126.1 (C8); 124.9 (C14); 124.4 (C8); 101.8 (C10); 93.6 (C29); 93.2 (C12); 83.3 (C2); 79.2 (C28); 79.0 (two coalesced lines, C33 and C16); 76.3 (C17); 69.6 (C35 and C36); 69.9 (η^5^-C_5_H_5_); 65.7 (C21); 64.7 (C32 and C34); 60.1 (C5); 58.4 (C27); 52.9 (C7); 51.4 (C25); 50.5 (C3); 43.4 (C6); 42.5 (C20); 39.0 (C26); 30.4 (C19); 29.6 (C30); 21.1 (C24); 8.1 (C18). Elemental composition: C_39_H_43_FeN_3_O_7_, HRMS: calculated *m*/*z*: 722.2523, measured *m*/*z*: 722.2508, mass error: 2.1 ppm (M+H^+^).

### 3.6. Synthesis of Chalcone-Containing Hybrids (***36*** and ***37***) by Sonogashira Coupling

The mixture of 600 mg of crude silyl-protected acetylene **30** (in pure form, 1 mmol is 550 mg), TBAF (523 mg, 2 mmol), the appropriate chalcone (**34**, 424 mg, 1 mmol) or **35** (442 mg, 1 mmol), CuI (38 mg, 0.2 mmol,), PdCl_2_(PPh_3_)_2_ (70 mg, 0.1 mmol), and DIPEA (0.53 mL, 390 mg, 3 mmol) was dissolved in DMF (20 mL). The mixture was stirred for 24 h at room temperature under an argon atmosphere and then poured into water. The precipitate was filtered off, washed with water (5 × 10 mL), dried, extracted with DCM (4 × 15 mL), and filtered off again. The organic solution was washed with water (1 × 30 mL) and brine, then dried over Na_2_SO_4_. After evaporation of the solvent, the solid residue was subjected to column chromatography on silica using solvent mixture DCM:MeOH (15:1) as eluent and crystallized from Et_2_O to obtain the pure product.

#### 3.6.1. Methyl (3aR,3a^1^R,4R,5S,5aR,10bR)-4-Acetoxy-3a-ethyl-5-hydroxy-8-methoxy-6-methyl-9-((4-((E)-3-oxo-3-(3,4,5-trimethoxyphenyl)prop-1-en-1-yl)phenyl)ethynyl)-3a,3a^1^,4,5,5a,6,11,12-octahydro-1H-indolizino[8,1-cd]carbazole-5-carboxylate (**36**)


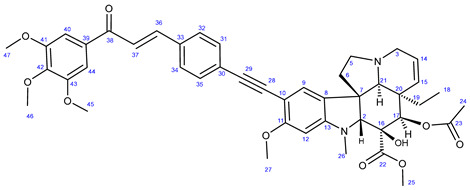


Light yellow powder. Mp: 149–150 °C; yield: 185 mg (24%) ^1^H-NMR (DMSO-*d*_6_): 8.87 (s, 1H, OH on C16); 7.98 (d, *J* = 16.2 Hz, 1H, H37); 7.94 (d, *J* = 8.2 Hz, 2H, H32 and H34); 7.76 (d, *J* = 16.2 Hz, 1H, H36); 7.51 (d, *J* = 8.2 Hz, 2H, H31 and H35); 7.45 (s, 2H, H40 and H44); 7.38 (s, 1H, H9); 6.26 (s, 1H, H12); 5.84 (ddd, *J* = 9.8 Hz, 4.8 Hz and 2.0 Hz, 1H, H14); 5.13 (s, 1H, H17); 5.11 (d, *J* = 9.8 Hz, 1H, H15); 3.91 (s, 6H, H45 and H47); 3.79 (s, 3H, H46); 3.76 (s, 3H, H27); 3.64 (s, 3H, H25); 3.57 (s, 1H, H2); 3.42 (m, 1H, H3/A); 3.30 (m, 1H, H5/A); 2.91 (br d, *J* = 16.2 Hz, 1H, H3/B); 2.71 (s, 1H, H21); 2.60 (m, 1H, H5/B); 2.26 (m, 2H, H6A and H6B); 2.59 (s, 3H, H26); 1.92 (s, 3H, H24); 1.48 (m, 1H, H19/A), 0.95 m, 1H; H19/B); 0.47 (t, *J* = 6.9 Hz, 3H, H18). ^13^C-NMR (DMSO-*d*_6_): 188.2 (C38); 172.6 (C22); 170.6 (C23); 162.3 (C11); 153.7 (C13); 153.4 (C41 and C43); 143.5 (C36); 142.5 (C42); 133.5 (two coalesced lines, C33 and C39); 131.6 (C31 and C35); 130.3 (C15); 129.8 (C32 and C34); 127.4 (C9); 126.1 (C8); 124.9 (C14); 124.4 (C8); 122.6 (C37); 106.2 (C40 and C44); 101.8 (C10); 93.4 (C12); 91.5 (C29); 91.3 (C28); 83.5 (C2); 79.0 (C16); 76.1 (C17); 65.4 (C21); 60.8 (C46); 60.1 (C5); 58.4 (C27); 56.8 (C45 and C47); 52.9 (C7); 51.4 (C25); 50.5 (C3); 43.4 (C6); 42.5 (C20); 39.1 (C26); 30.5 (C19); 21.1 (C24); 8.1 (C18). Elemental composition: C_45_H_48_N_2_O_10_, HRMS: calculated *m/z*: 777.3382, measured *m/z*: 777.34096, mass error: -3.6 ppm (M+H^+^).

#### 3.6.2. Methyl (3aR,3a^1^R,4R,5S,5aR,10bR)-4-Acetoxy-3a-ethyl-5-hydroxy-8-methoxy-6-methyl-9-((4-((E)-3-oxo-3-ferrocenylprop-1-en-1-yl)phenyl)ethynyl)-3a,3a^1^,4,5,5a,6,11,12-octahydro-1H-indolizino[8,1-cd]carbazole-5-carboxylate (**37**)


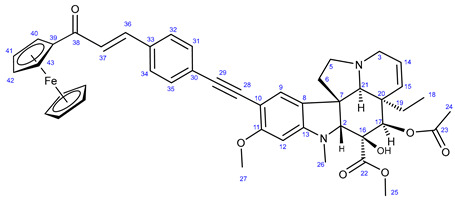


Orange powder. Mp: 163–165 °C (dec.); yield: 207 mg (26%) ^1^H-NMR (DMSO-*d*_6_): 8.85 (s, 1H, OH on C16); 7.89 (d, *J* = 8.2 Hz, 2H, H32 and H34); 7.63 (d, *J* = 16.2 Hz, 1H, H36); 7.50 (d, *J* = 8.2 Hz, 2H, H31 and H35); 7.47 (d, *J* = 16.2 Hz, 1H, H37); 7.32 (s, 1H, H9); 6.26 (s, 1H, H12); 5.85 (ddd, *J* = 9.8 Hz, 4.8 Hz and 2.0 Hz, 1H, H14); 5.12 (s, 1H, H17); 5.11 (d, *J* = 9.8 Hz, 1H, H15); 5.07 (t, *J* = 1.8 Hz, 2H, H40 and H43); 4.68 (t, *J* = 1.8 Hz, 2H, H41 and H42); 4.23 (s, 5H, η^5^C_5_H_5_); 3.76 (s, 3H, H27); 3.65 (s, 3H, H25); 3.57 (s, 1H, H2); 3.43 (m, 1H, H3/A); 3.30 (m, 1H, H5/A); 2.91 (br d, *J* = 16.2 Hz, 1H, H3/B); 2.71 (s, 1H, H21); 2.60 (m, 1H, H5/B); 2.26 (m, 2H, H6A and H6B); 2.59 (s, 3H, H26); 1.92 (s, 3H, H24); 1.49 (m, 1H, H19/A), 0.94 m, 1H; H19/B); 0.48 (t, *J* = 6.9 Hz, 3H, H18). ^13^C-NMR (DMSO-*d*_6_): 192.4 (C38); 172.7 (C22); 170.6 (C23); 162.3 (C11); 153.7 (C13); 153.4 (C41 and C43); 143.5 (C36); 142.5 (C42); 139.4 (C36); 134.6 (C33); 131.6 (C31 and C35); 130.3 (C15); 129.4 (C32 and C34); 127.4 (C9); 126.4 (C8); 125.6 (C30); 124.9 (C14); 124.6 (C37); 124.4 (C8); 101.8 (C10); 93.4 (C12); 91.5 (C29); 91.3 (C28); 83.5 (C2); 81.2 (C39); 79.0 (C16); 76.2 (C17); 73.3 (C41 and C42); 70.3 (η^5^C_5_H_5_); 65.4 (C21); 60.8 ((C46); 60.1 (C5); 58.4 (C27); 56.8 (C45 and C47); 52.9 (C7); 51.4 (C25); 50.5 (C3); 43.4 (C6); 42.4 (C20); 39.0 (C26); 30.6 (C19); 21.1 (C24); 8.2 (C18). Elemental composition: C_46_H_46_FeN_2_O_7_, HRMS: calculated *m*/*z*: 795.2727, measured *m*/*z*: 795.27252, mass error: 0.2 ppm (M+H^+^).

### 3.7. Synthesis of Reference Chalcone-Containing Hybrids (***36a*** and ***37a***) by Sonogashira Coupling

The mixture of phenylacetylene (102 mg, 1mmol), the appropriate iodochalcone (**34**, 424 mg, 1 mmol) or **35** (442 mg, 1 mmol), CuI (38 mg, 0.2 mmol,), PdCl_2_ (PPh_3_)_2_ (70 mg, 0.1 mmol), and DIPEA (0.53 mL, 390 mg, 3 mmol) was dissolved in DMF (20 mL). The mixture was stirred for 24 h at room temperature under an argon atmosphere then poured into water. The precipitate was filtered off, washed with water (5 x 10 mL), dried, extracted with DCM (4 x 15 mL), and filtered off again. The organic solution was washed with water (1 x 30 mL) and brine, then dried over Na_2_SO_4_. After evaporation of the solvent, the solid residue was subjected to column chromatography on silica using solvent mixture DCM:MeOH (15:1) as eluent and crystallized from Et_2_O to obtain the pure product.

#### 3.7.1. (E)-3-(4-(Phenylethynyl)phenyl)-1-(3,4,5-trimethoxyphenyl)prop-2-en-1-one (**36a**)


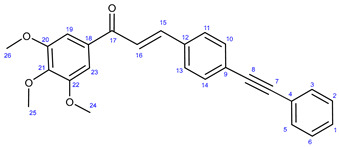


Light yellowish solid. Mp: 138–140 °C; yield: 316 mg (79%) ^1^H-NMR (DMSO-*d*_6_): 7.82 (d, *J* = 16.2 Hz, 1H, H15); 7.65 (d, *J* = 8.2 Hz, 2H, H11 and H13); 7.59 (d, *J* = 8.2 Hz, 2H, H10 and H14); 7.57 (m, 2H, H3 and H5); 7.51 (d, *J* = 16.2 Hz, 1H, H16); 7.43–7.36 (m, 3H, H1, H2 and H6); 7.31 (s, 2H, H19 and H23); 3.98 (s, 6H, H24 and H26); 3.97 (s, 3H, H25). ^13^C-NMR (DMSO-*d*_6_): 189.0 (C17); 153.2 (C20 and C22); 143.8 (C15); 142.6 (C21); 134.7 (C12); 133.4 (C18); 131.7 (C2 and C6); 132.1 (C10 and C14); 128.6 (C1); 128.44 (C3 and C5); 128.40 (C11 and C13); 125.5 (C9); 131.7 (C2 and C6); 122.9 (C4); 122.2 (C16); 106.2 (C19 and C23); 91.8 (C8); 89.1 (C7); 61.0 (C25); 56.5 (C24 and C26). Elemental composition: C_26_H_22_O_4_, HRMS: calculated *m*/*z*: 399.1591, measured *m/z*: 399.15923, mass error: −0.3 ppm (M+H^+^).

#### 3.7.2. (E)-1-(Ferrocenyl)-3-(4-(phenylethynyl)phenyl)prop-2-en-1-one (**37a**)


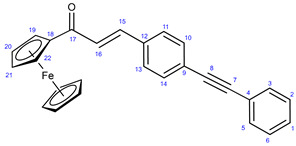


Light orange solid. Mp: 203–204 °C (dec.); yield: 308 mg (74%) ^1^H-NMR (DMSO-*d*_6_): 7.80 (d, *J* = 16.2 Hz, 1H, H15); 7.66 (d, *J* = 8.2 Hz, 2H, H11 and H13); 7.61 (d, *J* = 8.2 Hz, 2H, H10 and H14); 7.58 (m, 2H, H3 and H5); 7.43–7.36 (m, 3H, H1, H2 and H6); 7.16 (d, *J* = 16.2 Hz, 1H, H16); 4.95 (t, *J* = 1.8 Hz, 2H, H19 and H24); 4.63 (t, *J* = 1.8 Hz, 2H, H20 and H21); 4.26 (s, 5H, η^5^C_5_H_5_). ^13^C-NMR (DMSO-*d*_6_): 192.8 (C17); 140.0 (C15); 135.0 (C12); 132.1 (C10 and C14); 131.7 (C2 and C6); 128.6 (C1); 128.4 (C2 and C5); 128.2 (C11 and C13); 125.0 (C9); 123.5 (C16); 123.0 (C4); 91.8 (C8); 89.1 (C7); 80.6 (C18); 72.9 (C20 and C21); 70.2 (η^5^C_5_H_5_); 69.8 (C19 and C22). Elemental composition: C_27_H_20_FeO, HRMS: calculated *m*/*z*: 416.0858, measured *m*/*z*: 416.08557, mass error: 0.6 ppm (M^+^, Fe(II) oxidized to Fe(III)).

### 3.8. Determination of Antiproliferative Activities

The antiproliferative properties of a selected set of the prepared vindoline analogs were determined by the standard MTT [3-(4,5-dimethylthiazol-2-yl)-2,5-diphenyltetrazolium bromide] assay using MDA-MB-231, HeLa, A2780, and SH-SY5Y cell lines isolated from human breast, cervical, ovarian cancer, and neuroblastoma, respectively [68]. Non-cancerous human fibroblast MRC-5 cells were additionally used to characterize the cancer selectivity of the active tested agents. Cells were obtained from the European Collection of Cell Cultures (Salisbury, UK) and maintained in Eagle’s minimal essential medium (EMEM) supplemented with 10% fetal calf serum, 1% non-essential amino acids, and 1% antibiotic–antimycotic at 37 °C in a humidified atmosphere with 5% CO_2_. All media and supplements were purchased from Capricorn Scientific Ltd. (Ebsdorfergrund, Germany). Cells were seeded onto 96-well plates (5000/well), and after overnight incubation, the tested substances were added at two concentrations (10 or 30 µM). After incubation for 72 h under cell-culturing conditions, the MTT solution was added (20 µL of 5 mg/mL per well), and the medium was removed after 4 h. The generated formazan crystals were solubilized in 100 µM dimethylsulfoxide, and the absorbance was measured at 545 nm using a microplate reader (BMG Labtech, Ortenberg, Germany). Background-corrected values were used for further calculations. In the case of the test substances exhibiting higher than 50% growth inhibition at 10 µM on any cancer cell lines, the assays were repeated using a set of dilutions to obtain IC_50_ values. In the same cases, the compounds were tested against MRC-5 fibroblasts to characterize their cancer selectivity. Two independent experiments were performed with five parallel wells. Cisplatin (Ebewe Pharma GmbH, Unterach, Austria) was included as an additional reference agent. The IC_50_ values were calculated by fitting sigmoid concentration–response curves using GraphPad Prism 10.0 software (GraphPad Software, San Diego, CA, USA).

## 4. Conclusions

This contribution presents feasible synthetic pathways for the synthesis of the first representatives of alkyne-tethered vindoline-based hybrids as potential anticancer agents. The antiproliferative assays identified a trimethoxyphenyl-containing chalcone–vindoline hybrid (**36**) as a highly efficient and selective lead compound featuring a wide therapeutic window determined by its submicromolar activity against A2780 cells and a substantially decreased activity against MRC-5 fibroblast cells. Consequently, ovarian cancer might be considered a prioritized target of treatment with **36**, which merits more extended investigation to disclose its cellular targets and mechanism of action, paving the way for developing rationally designed follow-up molecules with enhanced potency in clinical applications.

## Figures and Tables

**Table 1:** Initial screening of the antiproliferative effect of the alkyne-tethered vindoline hybrids and reference compounds, including vindoline **1b**, measured using the investigated cells.

Compound	Conc. (µM)	Mean Growth Inhibition (%) ± SEM				
		MRC-5	MDA-MB-231	HeLa	A2780	SH-SY5Y
**21**	10	Not tested	<20	<20	<20	<20
	30		<20	21.41 ± 1.22	29.46 ± 1.03	<20
**22**	10	Not tested	<20	<20	24.69 ± 2.03	<20
	30		89.92 ± 0.65	43.95 ± 1.74	97.13 ± 0.40	94.91 ± 1.85
**23**	10	Not tested	<20	<20	<20	<20
	30		<20	<20	<20	<20
**24**	10	<20	47.84 ± 2.98	<20	75.74 ± 2.55	53.41 ± 0.89
	30	<20	80.37 ± 1.83	<20	88.05 ± 1.08	82.74 ± 2.85
**28**	10	Not tested	<20	<20	<20	<20
	30		<20	<20	49.19 ± 1.98	<20
**29**	10	Not tested	<20	<20	<20	<20
	30		23.99 ± 0.81	<20	68.02 ± 1.37	91.64 ± 1.10
**36**	10	87.09 ± 2.73	86.22 ± 0.79	89.62 ± 0.75	95.09 ± 0.50	89.00 ± 2.61
	30	88.90 ± 2.80	85.01 ± 0.87	89.98 ± 0.46	94.50 ± 0.35	89.10 ± 2.74
**36a**	10	46.14 ± 2.17	86.37 ± 0.71	90.55 ± 0.72	95.53 ± 0.34	90.01 ± 2.84
	30	89.88 ± 3.24	86.28 ± 0.47	90.29 ± 0.28	95.07 ± 0.27	91.67 ± 3.36
**37**	10	Not tested	<20	<20	<20	<20
	30		<20	<20	31.96 ± 2.40	<20
**37a**	10	27.48 ± 2.20	70.70 ±1.61	51.26 ± 0.79	57.17 ± 1.48	<20
	30	52.25 ± 2.01	80.99 ± 1.49	71.19 ± 2.86	81.63 ± 2.56	51.77 ± 3.06
**1b**	10	<20	<20	<20	<20	<20
	30	<20	<20	<20	<20	<20

**Table 2:** IC_50_ values obtained for the most active compounds identified in the initial viability tests.

Compound	IC_50_ (µM)				
	MRC-5	MDA-MB-231	HeLa	A2780	SH-SY5Y
**24**	n.d.	11.78	n.d.	5.62	10.33
**36**	2.55	1.15 (2.22 ^a^)	1.57 (1.62 ^a^)	**0.60 (4.25 ^a^)**	1.26 (2.02 ^a^)
**36a**	10.92	4.22 (2.59 ^a^)	4.54 (2.40 ^a^)	3.26 (3.35 ^a^)	5.60 (1.95 ^a^)
**37a**	27.42	7.79 (3.52 ^a^)	11.47 (2.39 ^a^)	8.84 (3.10 ^a^)	29.16 (0.94 ^a^)
**Cisplatin ^b^**	4.51 ^b^	19.10 ^b^ (0.24 ^a^)	12.40 ^b^ (0.36 ^a^)	1.30 ^b^ (3.47 ^a^)	3.79 (1.19 ^a^)

^a^ Selectivity indices (IC_50_ on MRC-5/IC_50_ on the cancer cell). ^b^ Reference drug. Values are from reference [64] except for unpublished IC_50_ measured for SH-SY5Y.

## Data Availability

The data generated and analyzed during our research are not available in any public database or repository but will be shared by the corresponding author upon reasonable request.

## Supplementary Materials

The following supporting information can be downloaded at https://www.mdpi.com/article/10.3390/ijms25137428/s1.
