# Supplementary material for: Synthesis and Antiproliferative Effect of New Alkyne-Tethered Vindoline Hybrids Containing Pharmacophoric Fragments
Source: Int J Mol Sci. 2024 Jul 6;25(13):7428. doi: 10.3390/ijms25137428 (PMC11242353; doi:10.3390/ijms25137428)
Supplement: Supplementary file 1 [file ijms-25-07428-s001.zip › ijms-3058134-supplementary.pdf]

International Journal of *Molecular Sciences*

MDPI

Synthesis and Antiproliferative Effect of New Alkyne-Tethered Vindoline Hybrids Containing Pharmacophoric Fragments

Etelka Ferenczi [1,2], Péter Keglevich [3], Bizhar Ahmed Tayeb [4], Renáta Minorics [4], Dávid Papp [2,5], Gitta Schlosser [5], István Zupkó [4], László Hazai [3] and Antal Csámpai [1,*]

[1] Department of Organic Chemistry, Eötvös Loránd University (ELTE), Pázmány P. sétány 1/A, H-1117 Budapest, Hungary; ferenczie@student.elte.hu
[2] Hevesy György PhD School of Chemistry, Pázmány P. sétány 1/A, H-1117 Budapest, Hungary; david.papp@ttk.elte.hu
[3] Department of Organic Chemistry and Technology, Faculty of Chemical Technology and Biotechnology, Budapest University of Technology and Economics, Műegyetem rkp. 3, H-1111 Budapest, Hungary; keglevich.peter@vbk.bme.hu (P.K.); hazai.laszlo@vbk.bme.hu (L.H.)
[4] Institute of Pharmacodynamics and Biopharmacy, University of Szeged, Eötvös u. 6, H-6720 Szeged, Hungary; drbat25@gmail.com (B.A.T.); kanizsaine.minorics.renata@szte.hu (R.M.); zupko.istvan@szte.hu (I.Z.)
[5] MTA-ELTE Lendület Ion Mobility Mass Spectrometry Research Group, Institute of Chemistry, ELTE Eötvös Loránd University, Pázmány Péter sétány 1/A, H-1117 Budapest, Hungary; gitta.schlosser@ttk.elte.hu
* Correspondence: antal.csampai@ttk.elte.hu; Tel.: +36-01-372-2500 (ext. 6591)

Supporting Information

Table of content

1 Copies of NMR spectra S2
1.1 NMR spectra of the novel propargylated pharmacophore fragments S2-S5
1.2 NMR spectra of the novel iodinated chalcones S6-S7
1.3 NMR spectra of the novel alkyne-tethered vindoline hybrids S8-S16
2 Copies of HRMS spectra S17
2.1 HRMS spectra of the novel alkyne-tethered vindoline hybrids S17-S20

1 Copies of NMR spectra

1.1 NMR spectra of the novel propargylated pharmacophore fragments

^{1}H-NMR of **10** (500 MHz, DMSO-d_6)

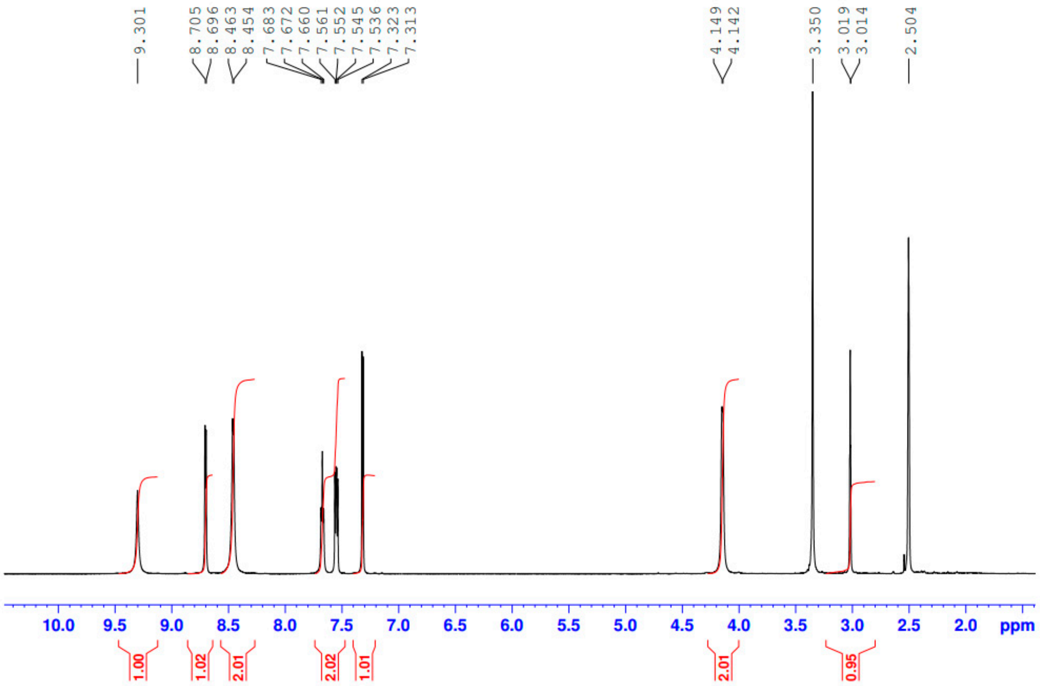

^{13}C-NMR of **10** (125 MHz, DMSO-d$_6$)

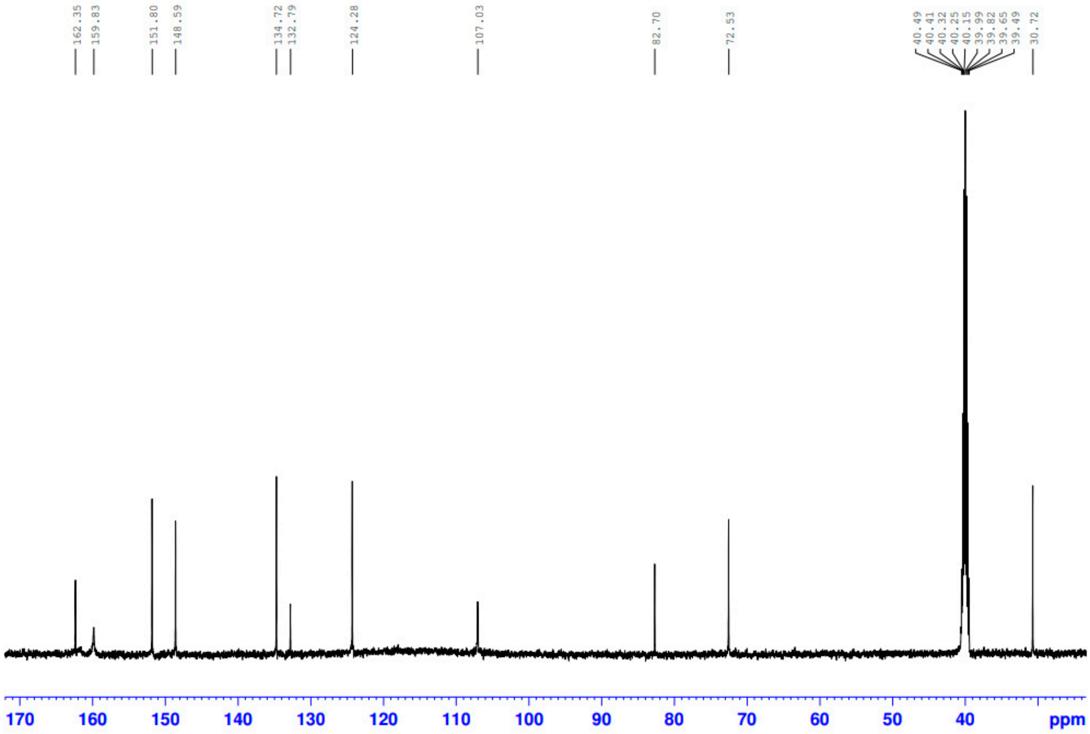

^{1}H-NMR of **15** (500 MHz, DMSO-d$_6$)

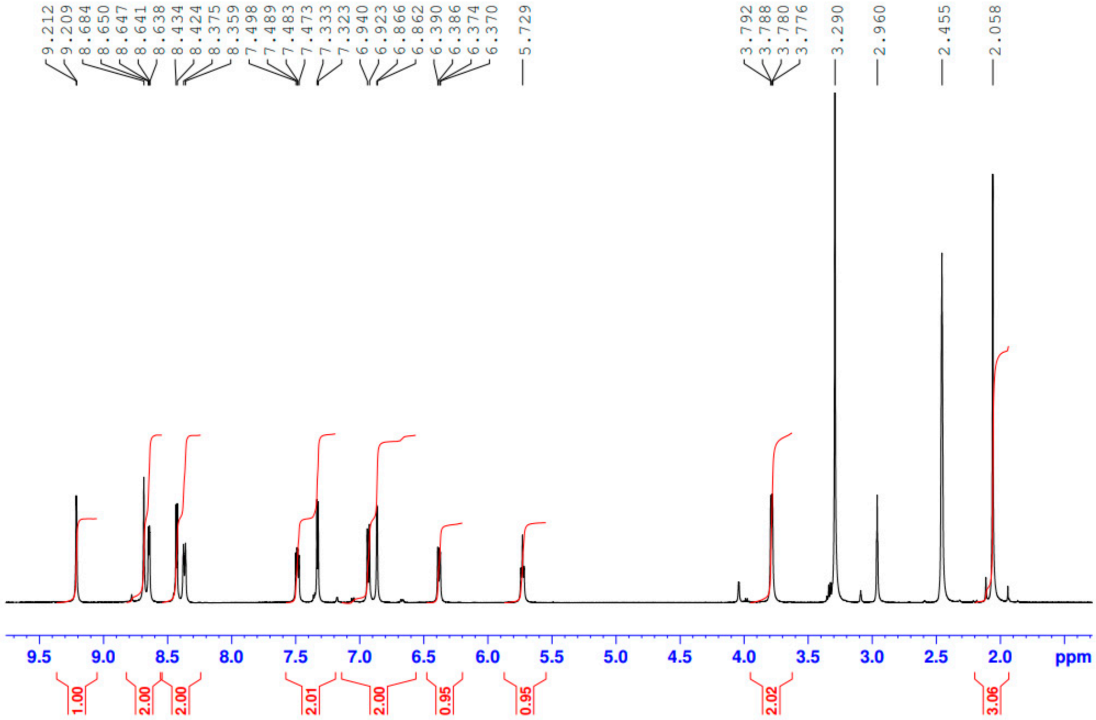

^{13}C-NMR of **15** (125 MHz, DMSO-d$_6$)

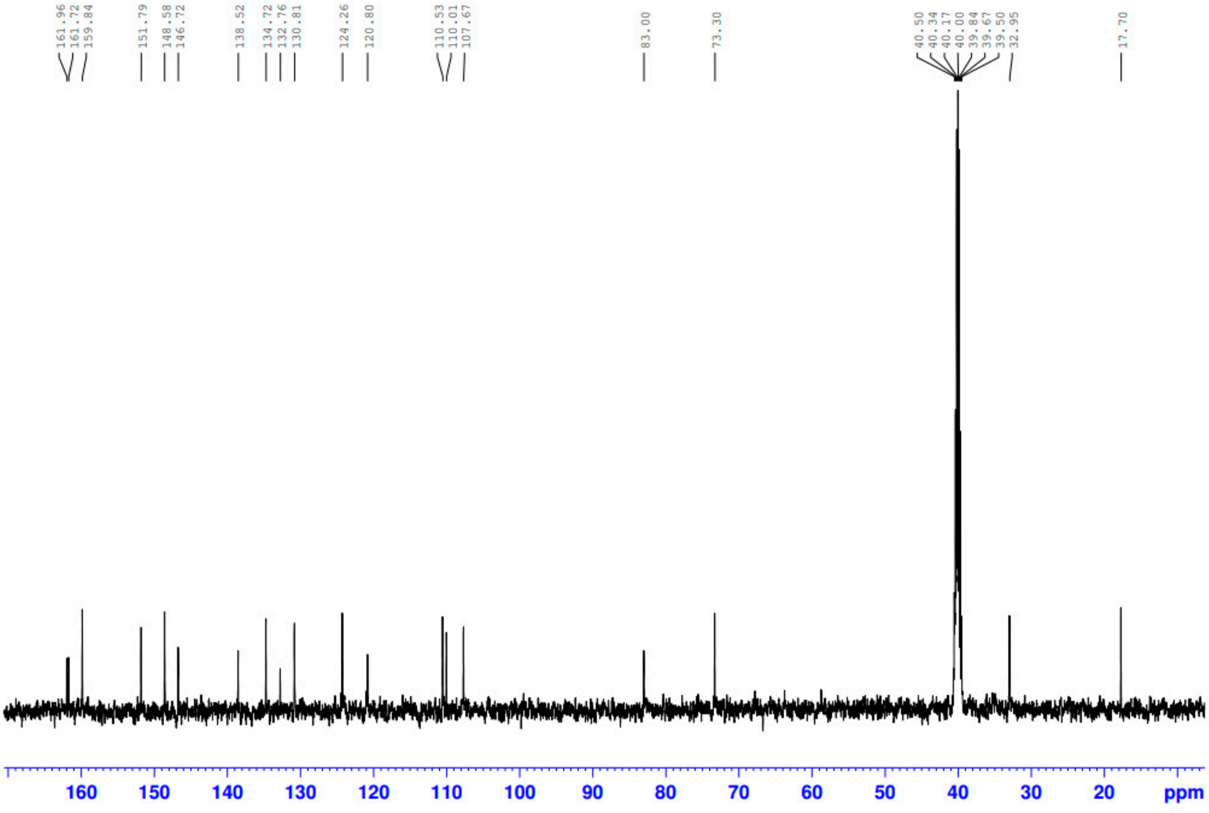

^{1}H-NMR of **19** (500 MHz, DMSO-d$_6$)

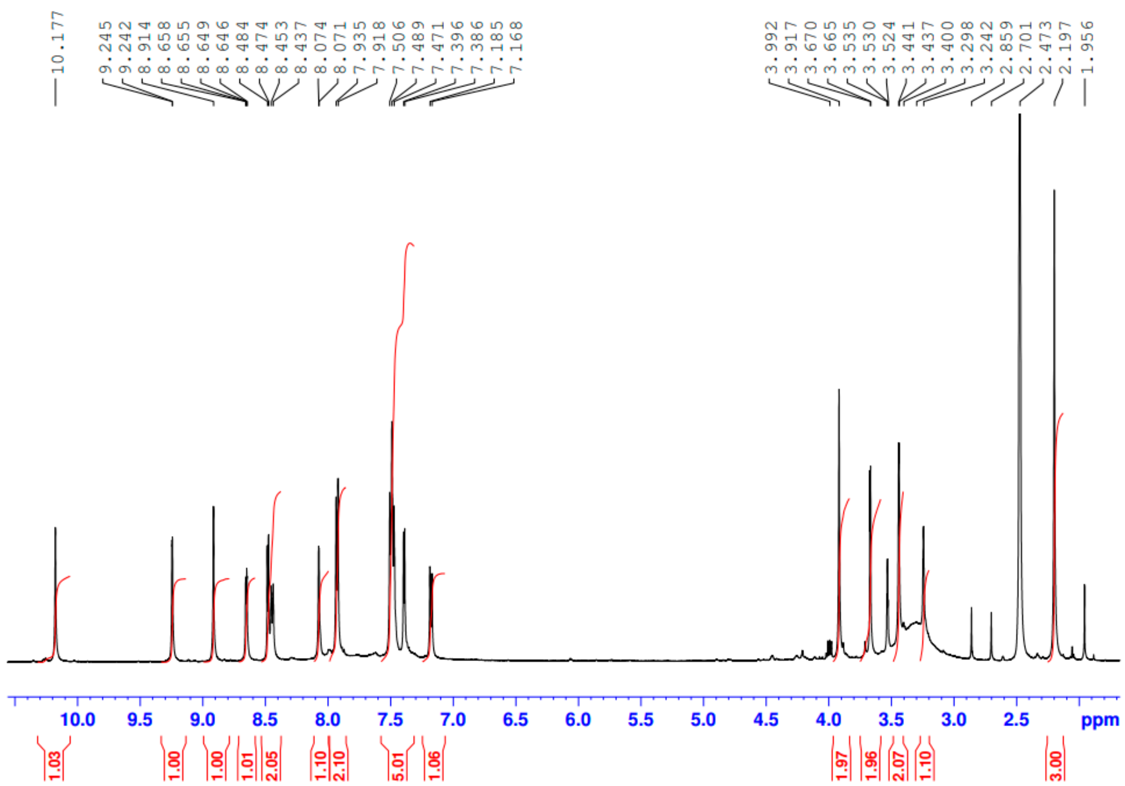

^{13}C-NMR of **19** (125 MHz, DMSO-d$_6$)

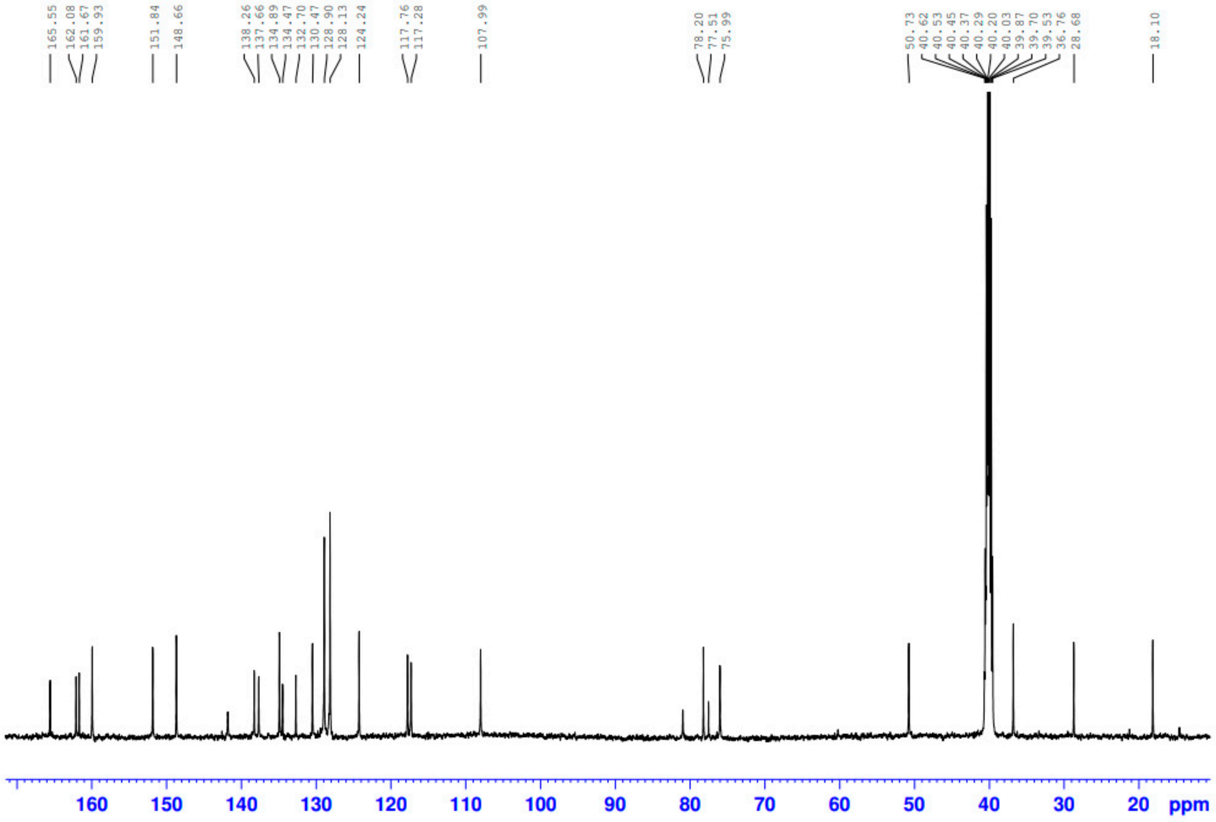

^{1}H-NMR of **27** (500 MHz, DMSO-d6)

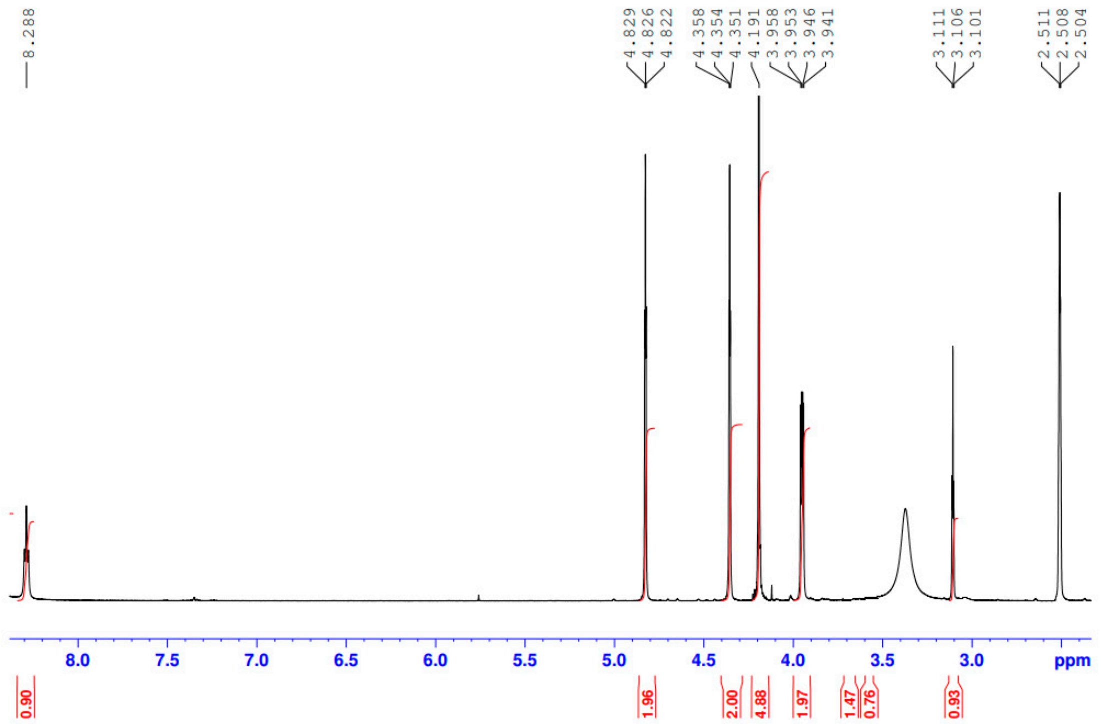

^{13}C-NMR of **27** (125 MHz, DMSO-d6)

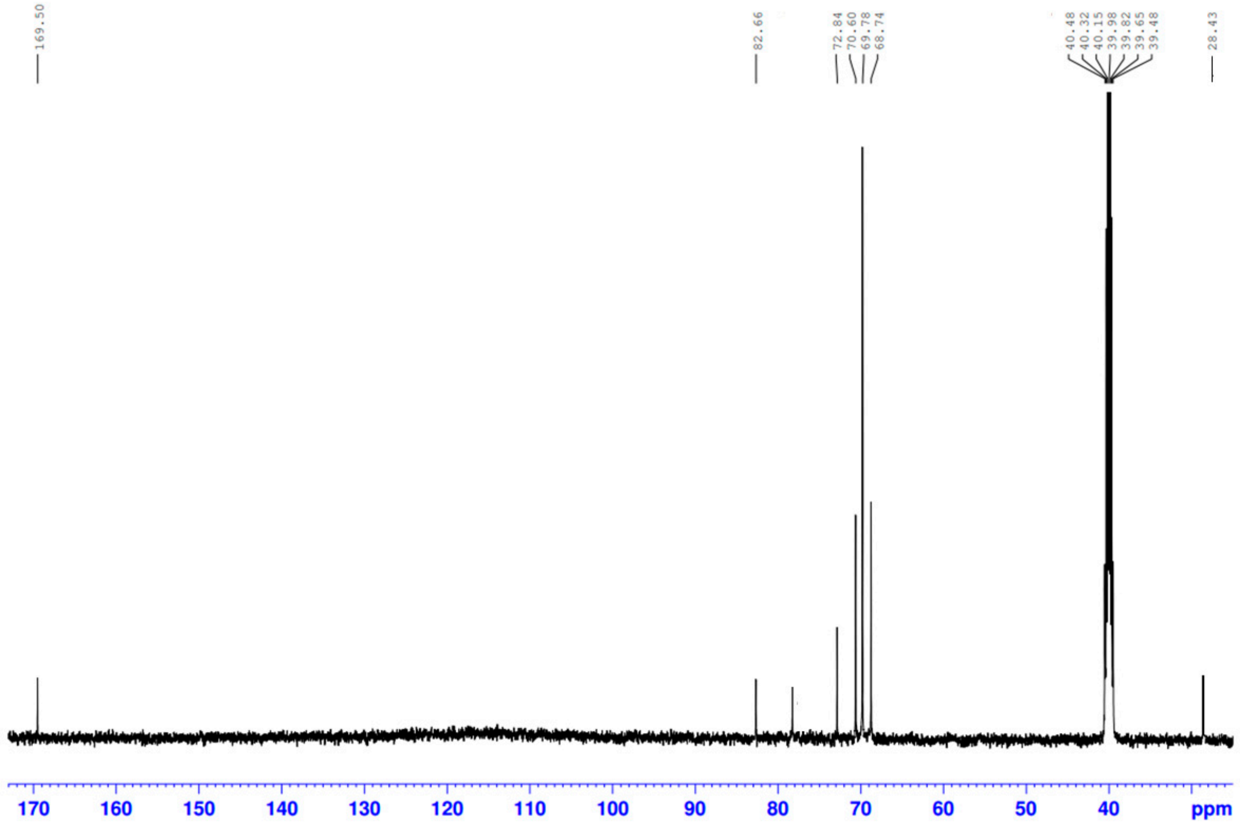

1.2 NMR spectra of the novel iodinated chalcones

^{1}H-NMR of **34** (500 MHz, DMSO-d$_6$)

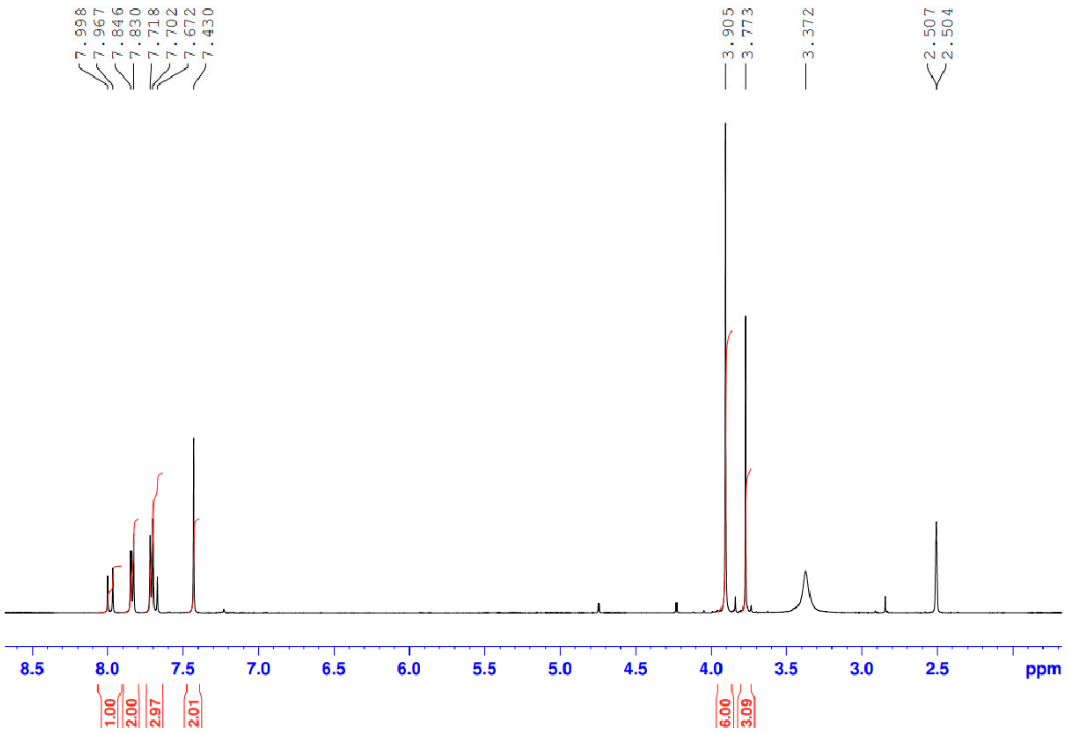

^{13}C-NMR of **34** (500 MHz, DMSO-d$_6$)

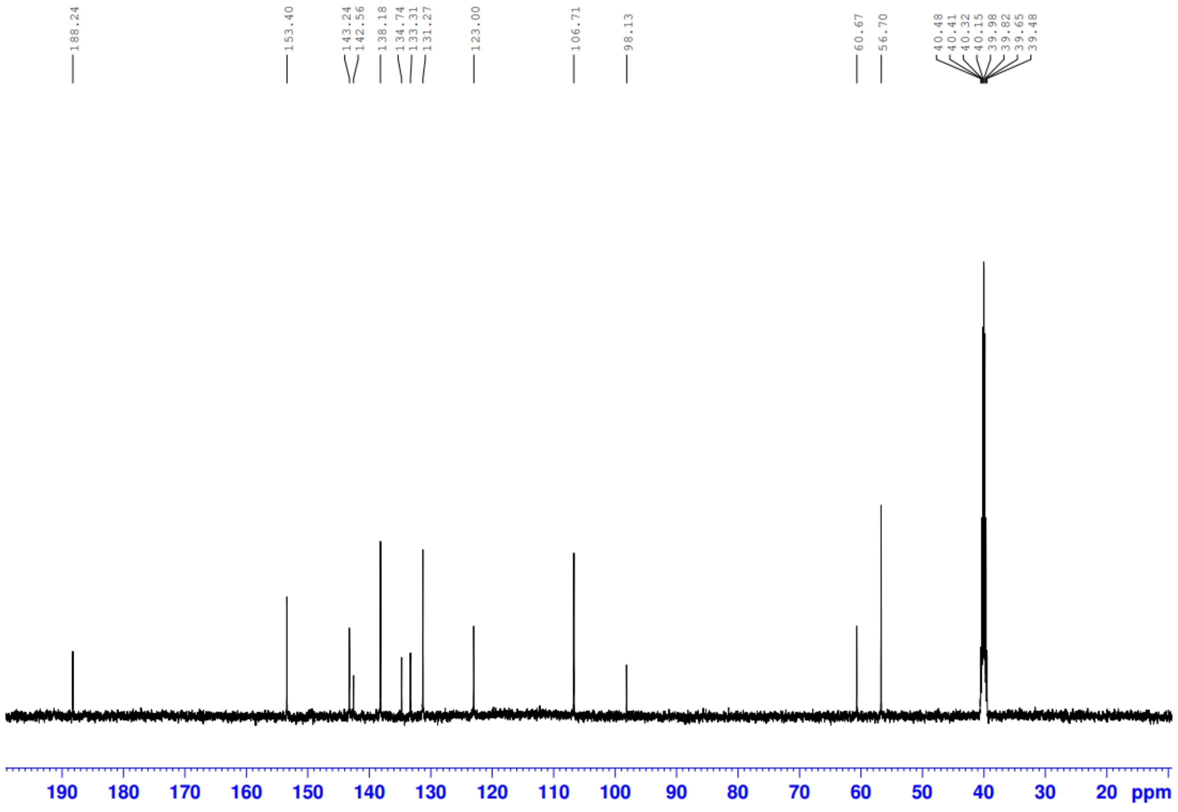

^{1}H-NMR of **35** (500 MHz, DMSO-d6)

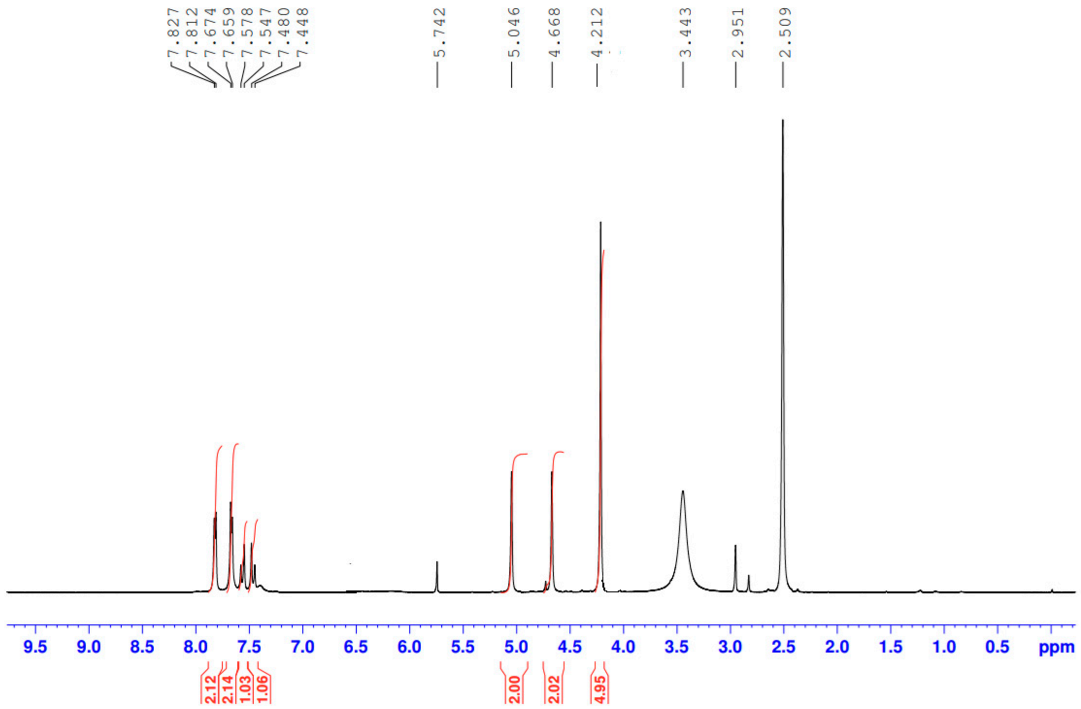

^{13}C-NMR of **35** (500 MHz, DMSO-d6)

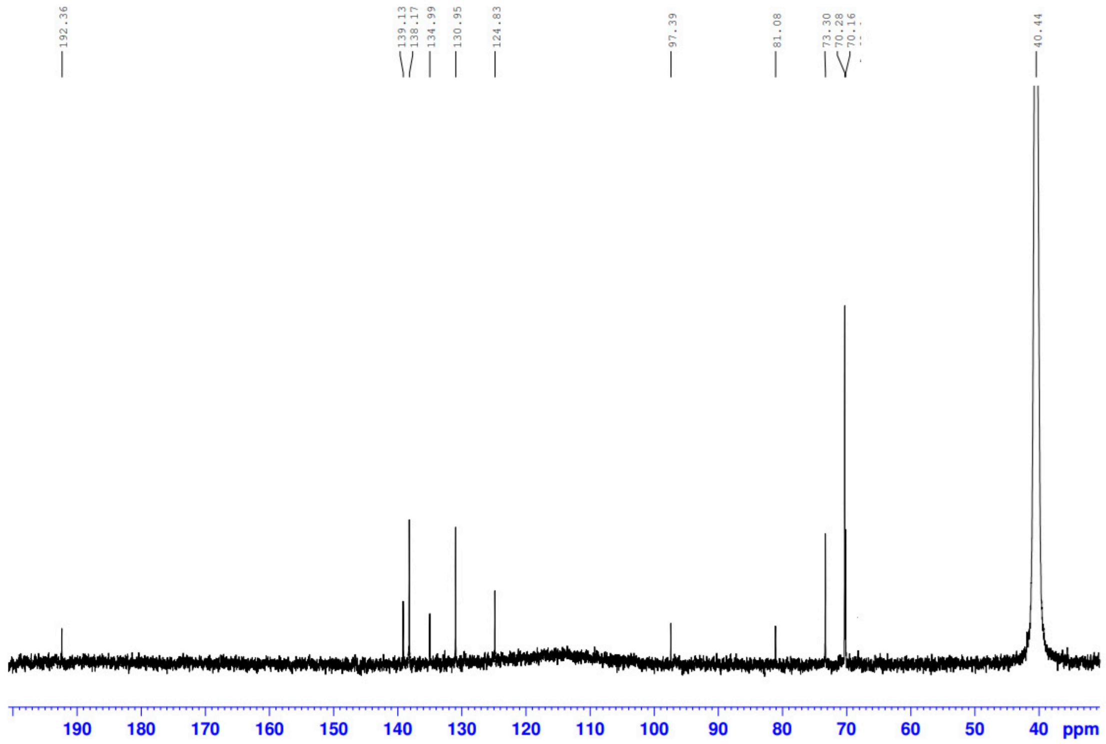

1.3 *NMR spectra of the novel alkyne-tethered vindoline hybrids*

^{1}H-NMR of **22** (500 MHz, DMSO-d_6)

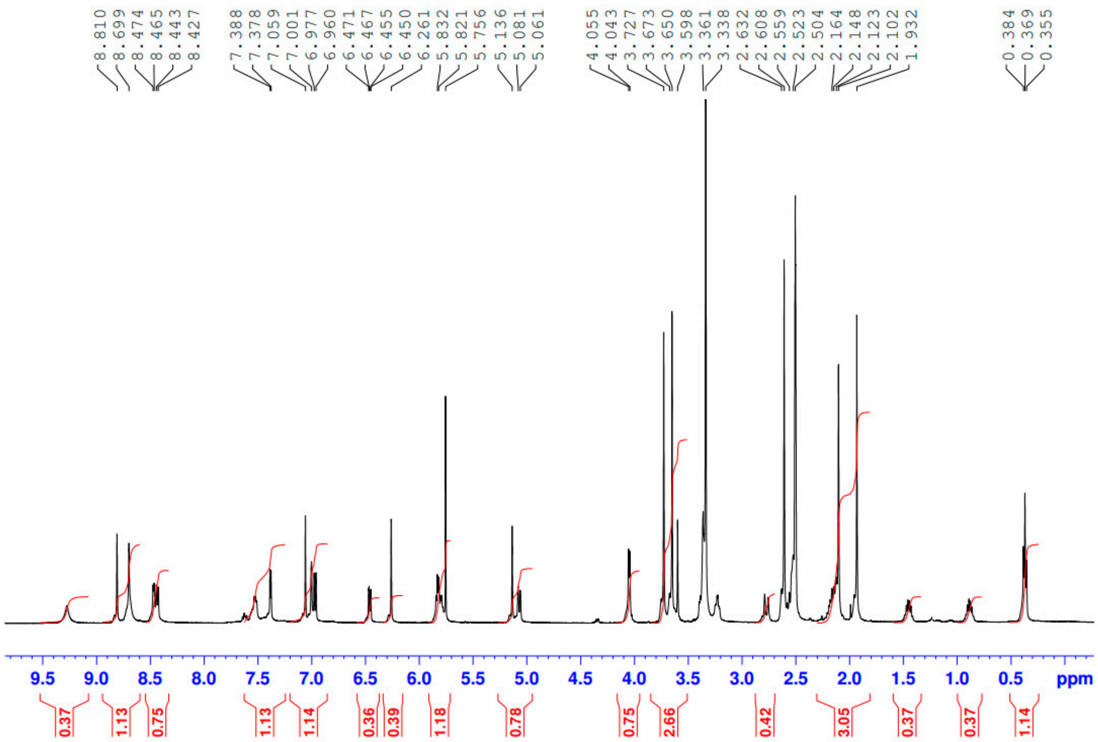

^{13}C-NMR of **22** (125 MHz, DMSO-d_6)

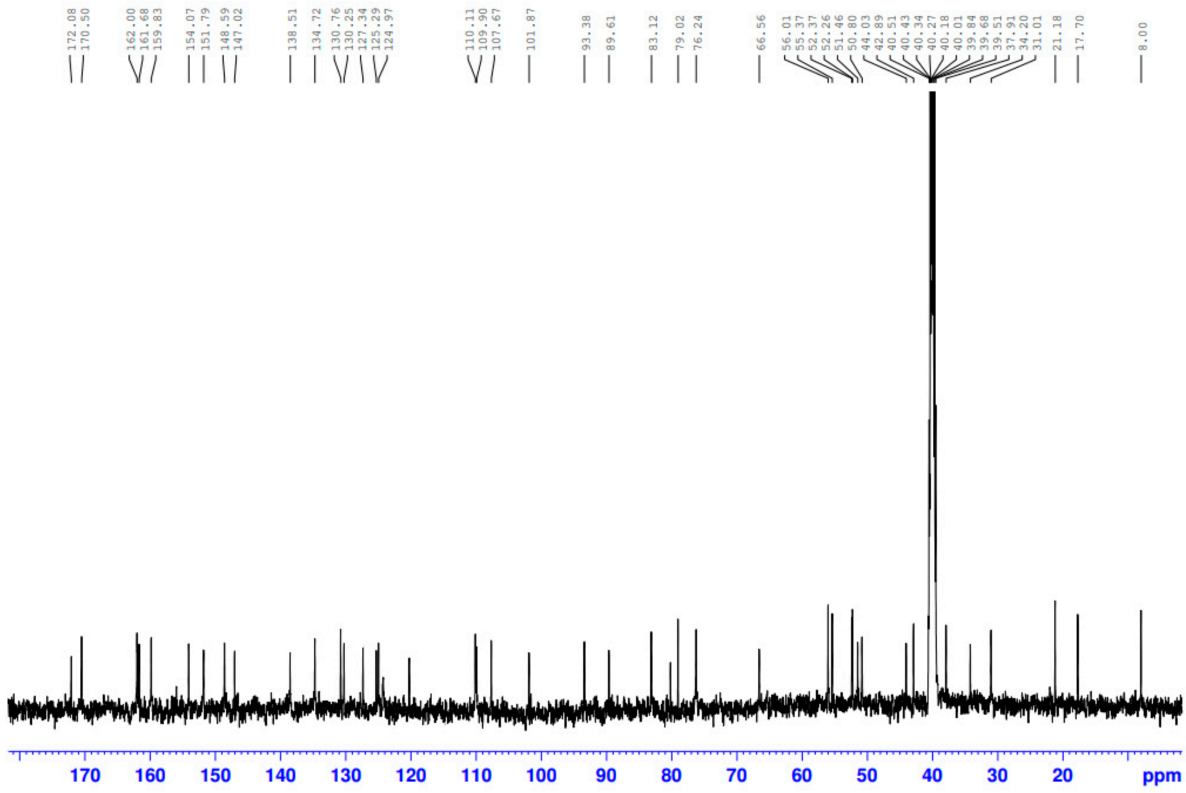

^{1}H-NMR of **23** (500 MHz, DMSO-d6)

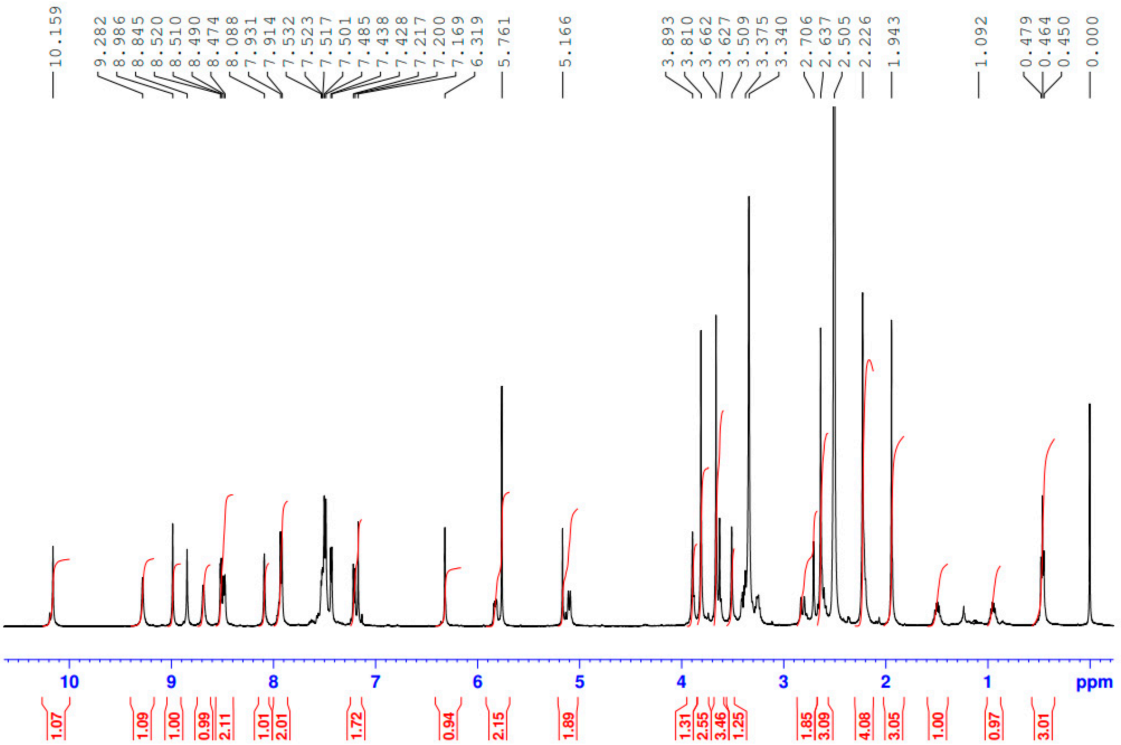

^{13}C-NMR of **23** (125 MHz, DMSO-d6)

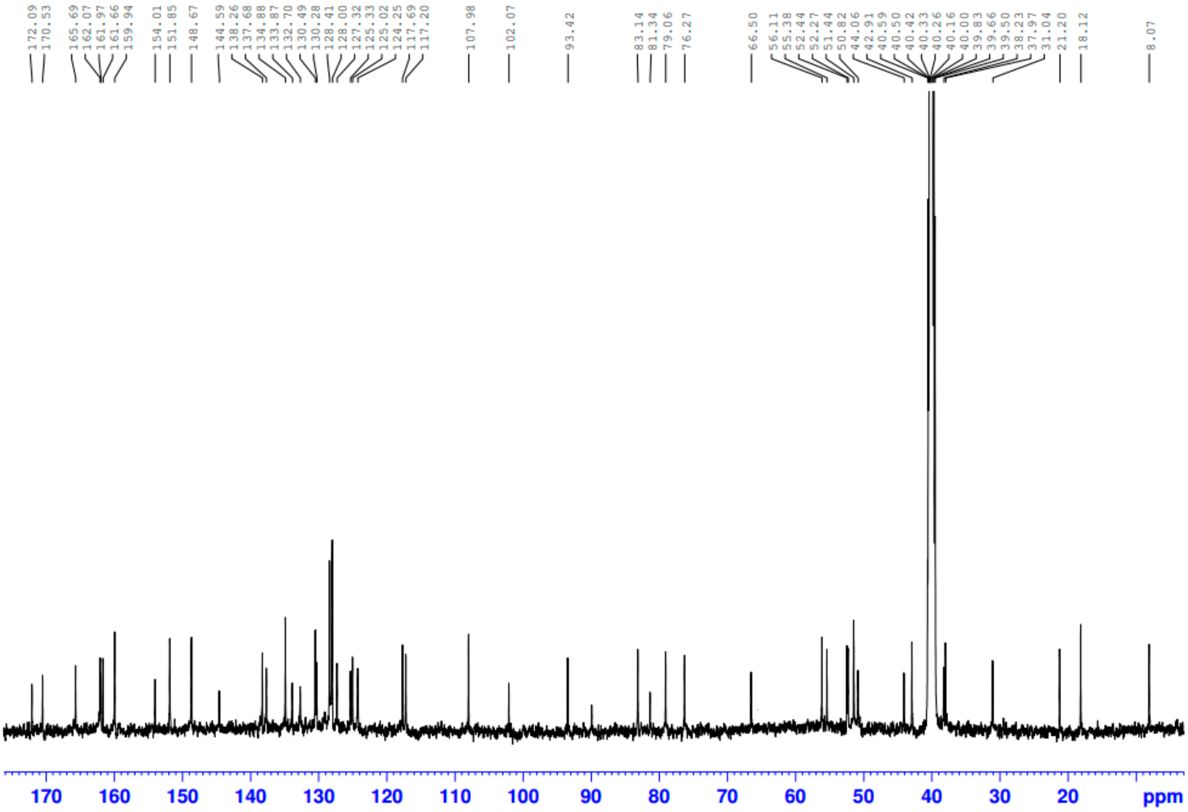

^{1}H-NMR of **24** (500 MHz, DMSO-d$_6$)

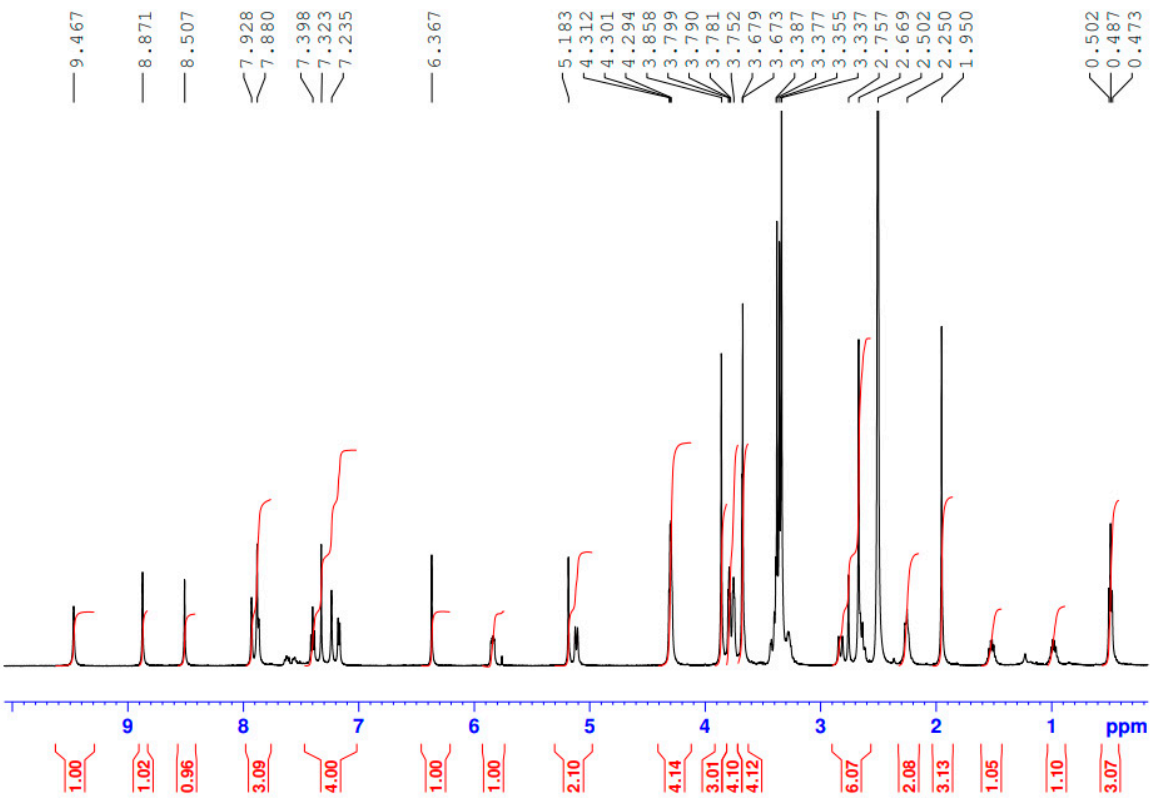

^{13}C-NMR of **24** (125 MHz, DMSO-d$_6$)

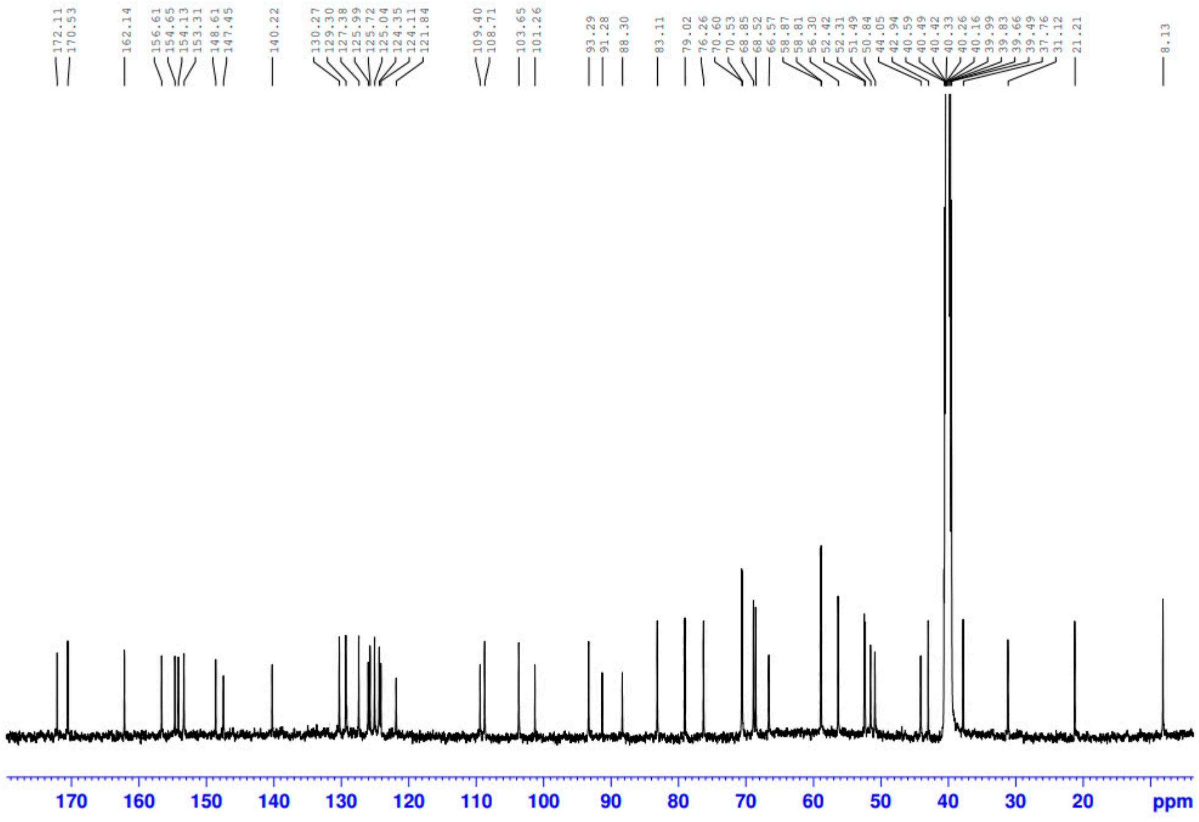

^{1}H-NMR of **28** (500 MHz, DMSO-d6)

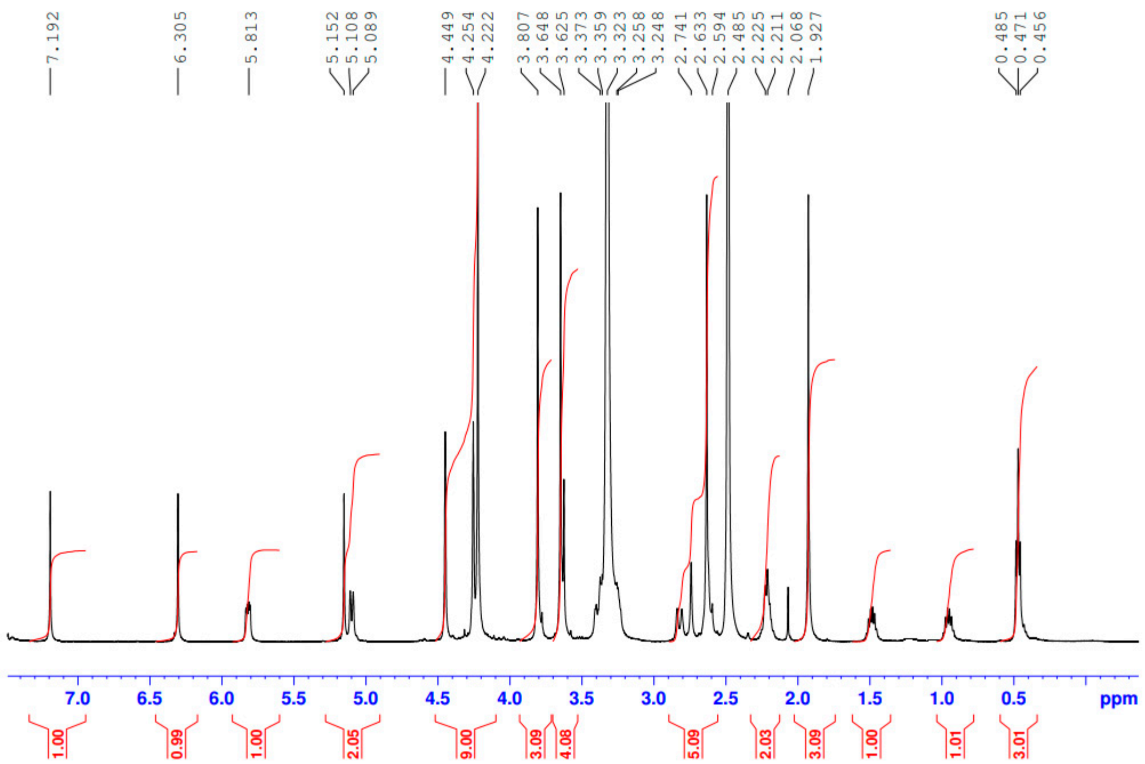

^{13}C-NMR of **28** (125 MHz, DMSO-d6)

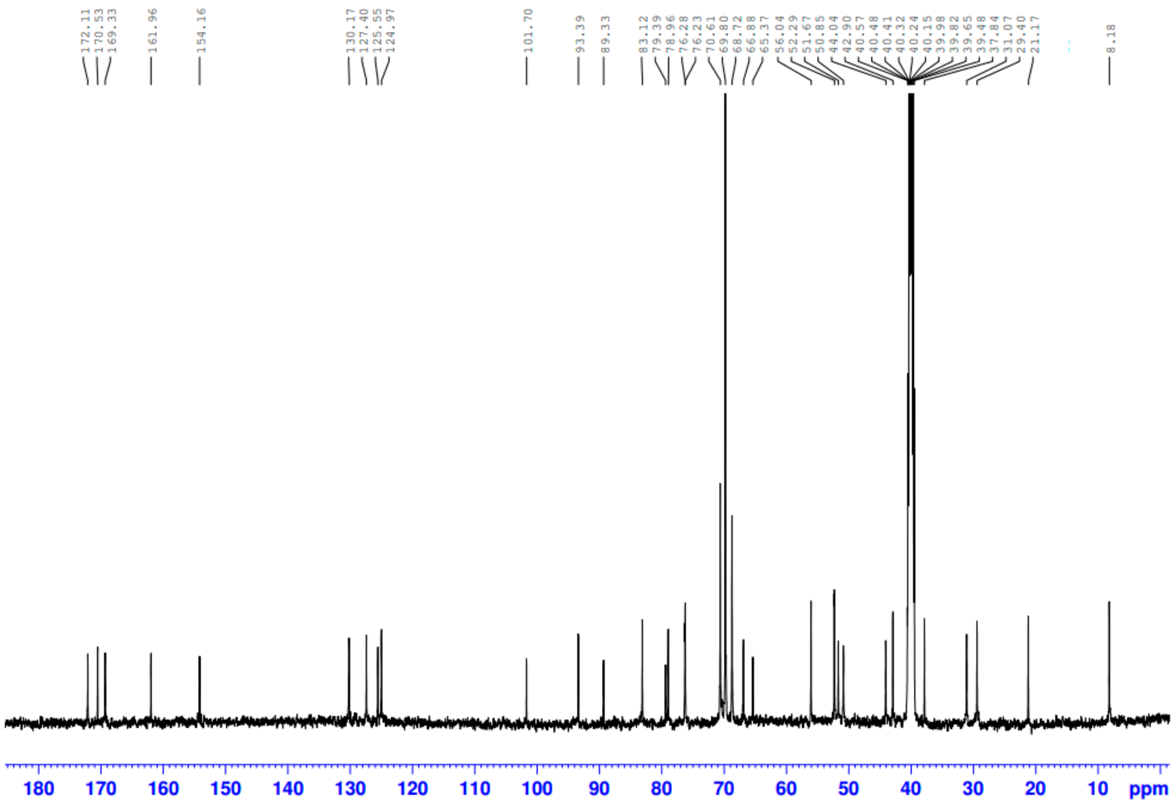

^{1}H-NMR of **29** (500 MHz, DMSO-d6)

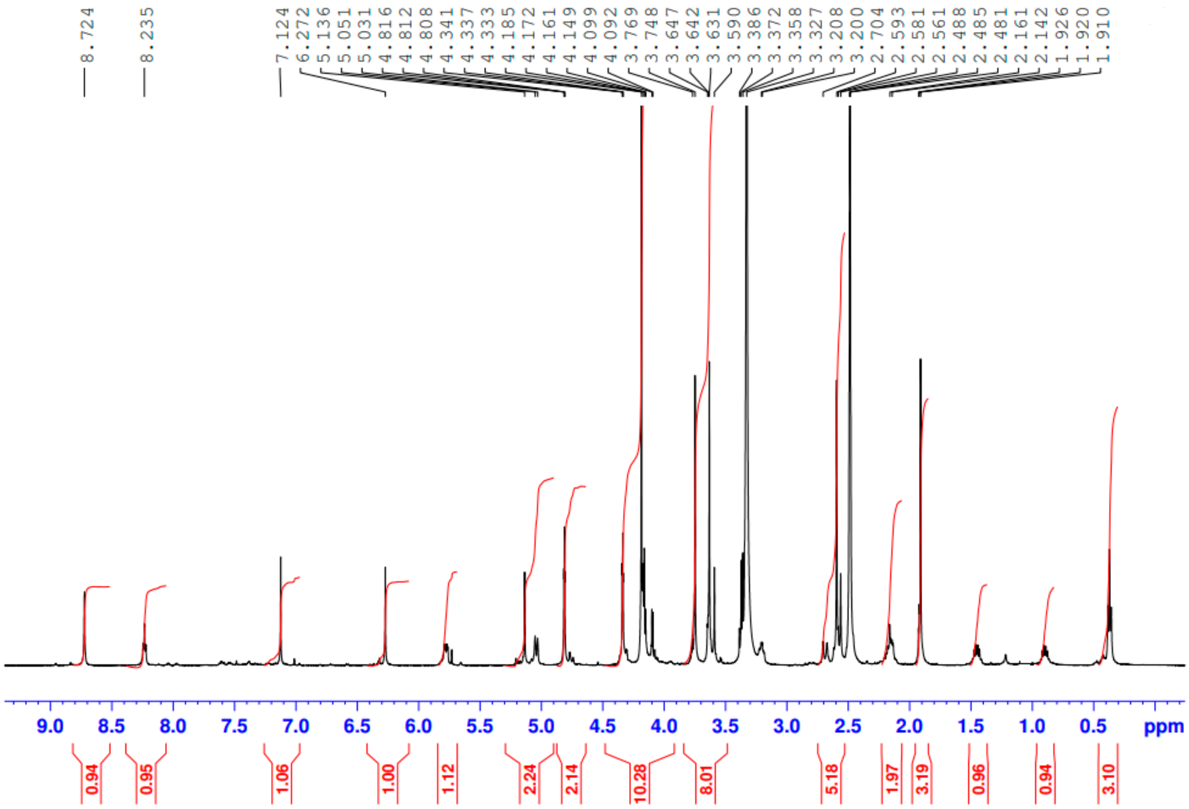

^{13}C-NMR of **29** (125 MHz, DMSO-d6)

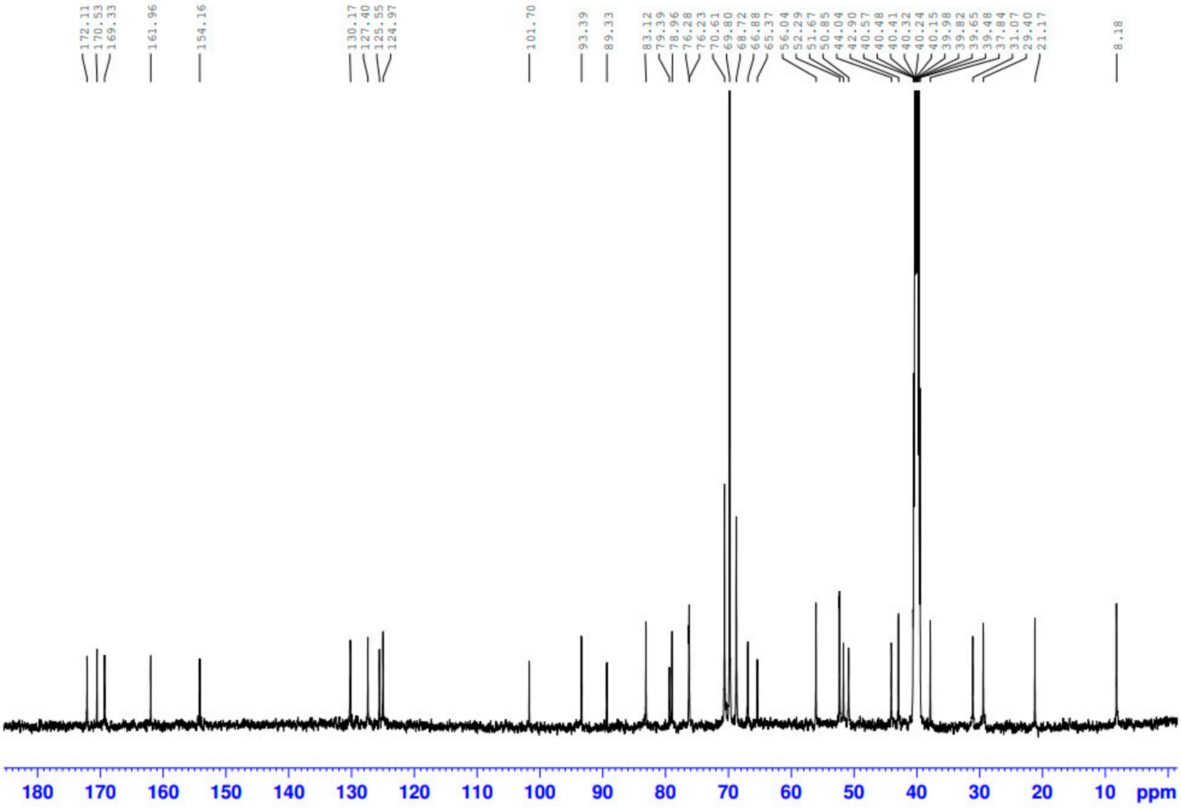

^{1}H-NMR of **36** (500 MHz, DMSO-d6)

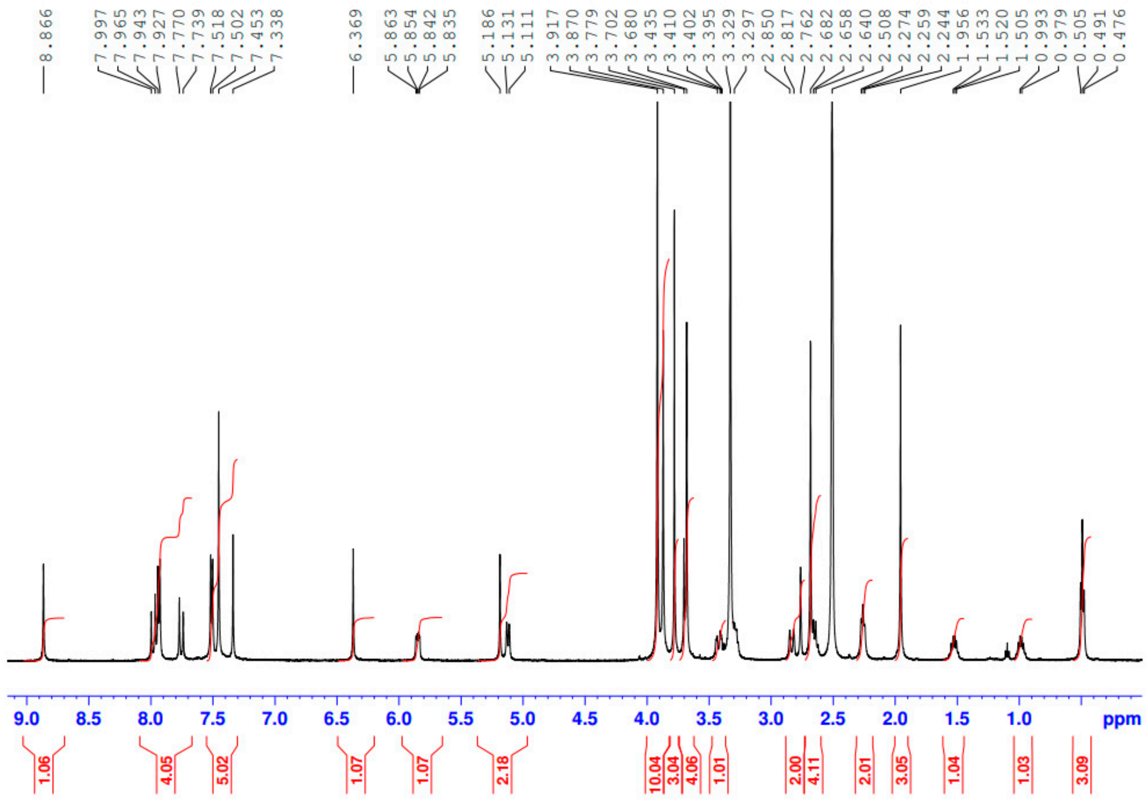

^{13}C-NMR of **36** (125 MHz, DMSO-d6)

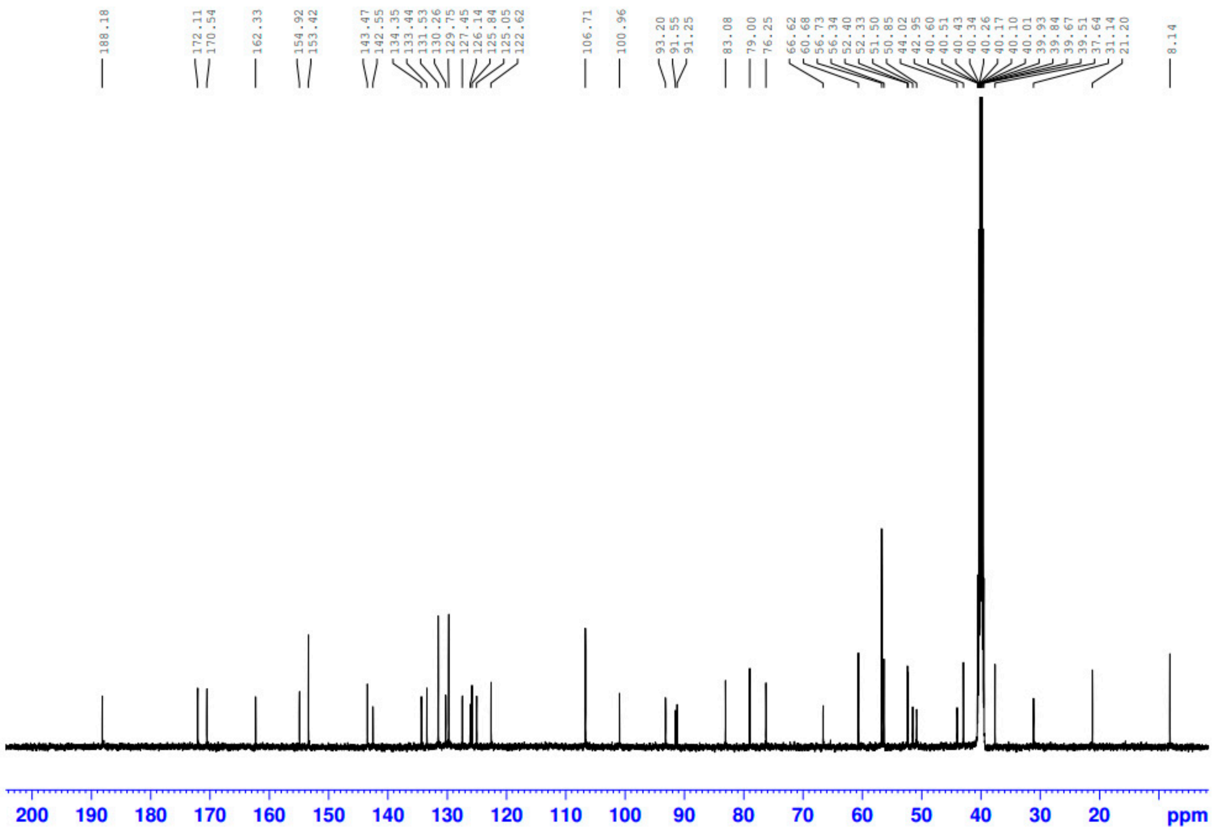

^{1}H-NMR of **37** (500 MHz, DMSO-d_6)

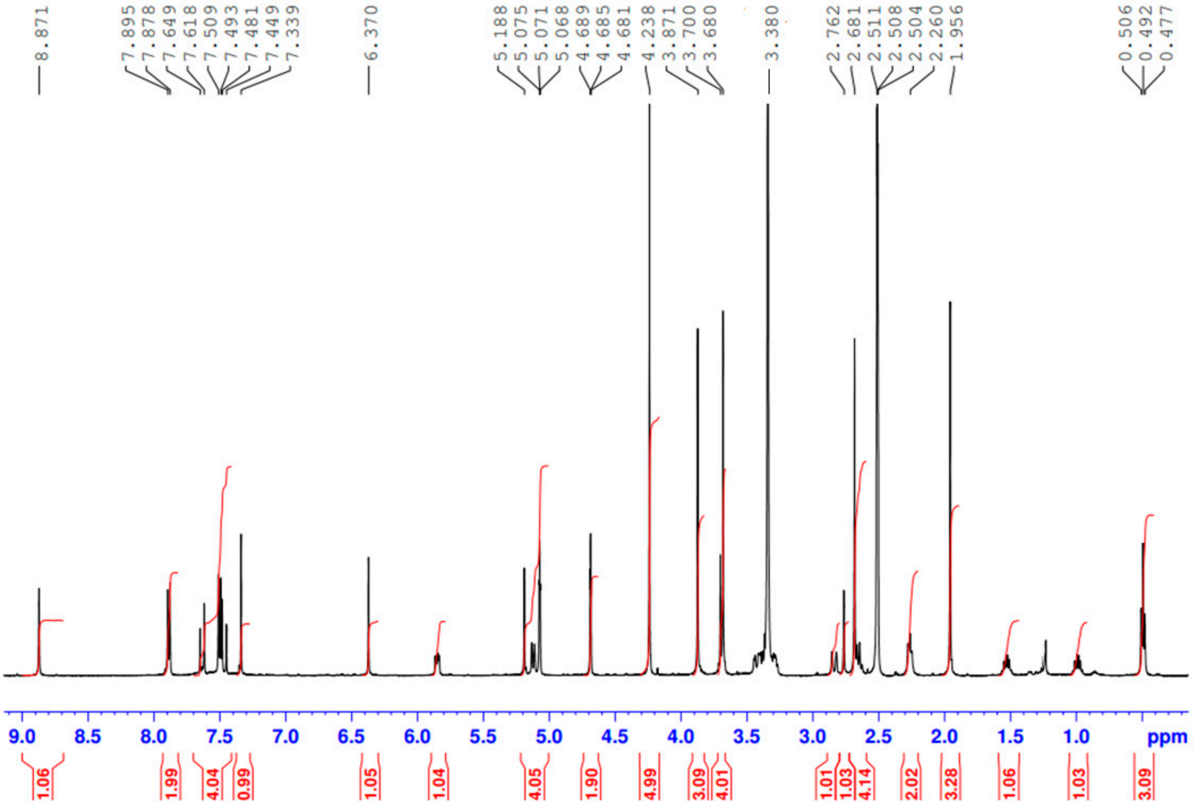

^{13}C-NMR of **37** (125 MHz, DMSO-d_6)

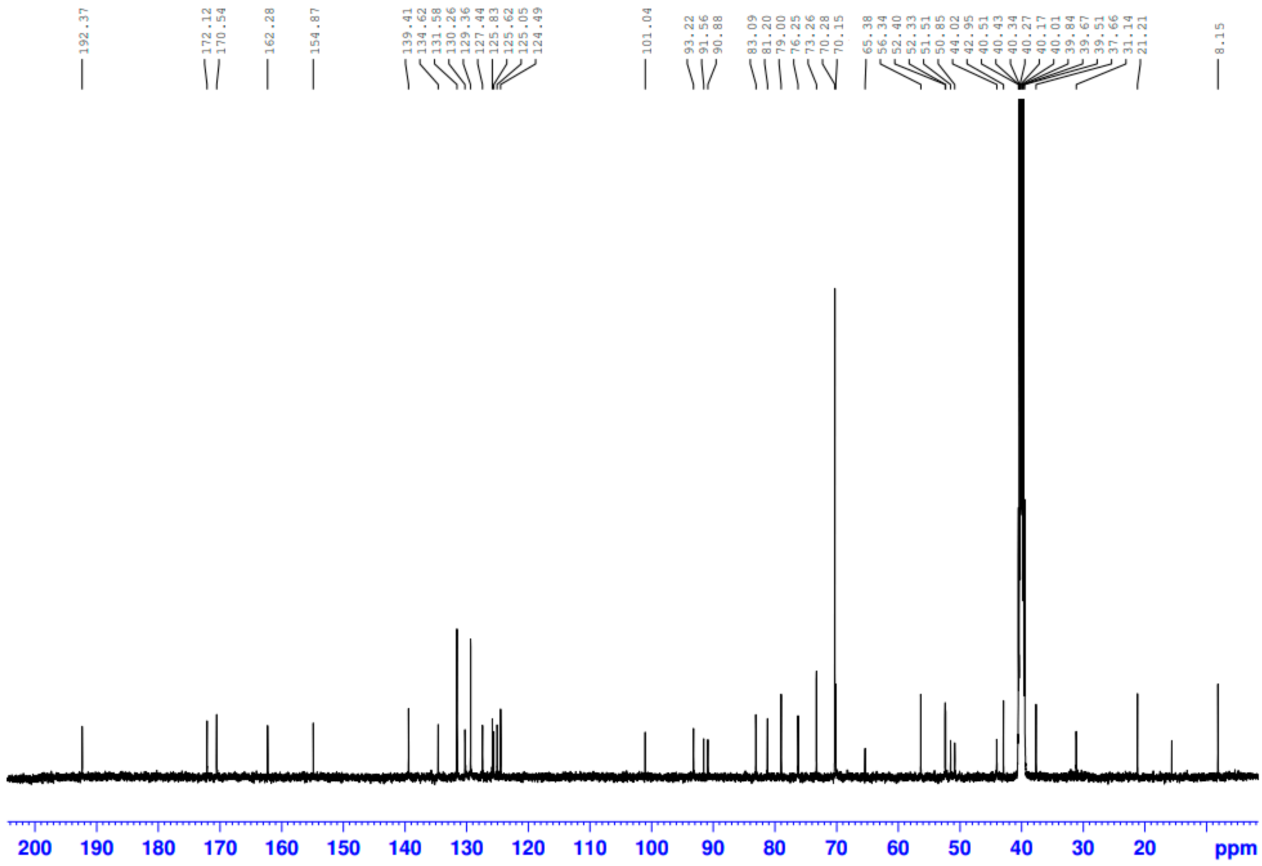

^{1}H-NMR of **36a** (500 MHz, DMSO-d6)

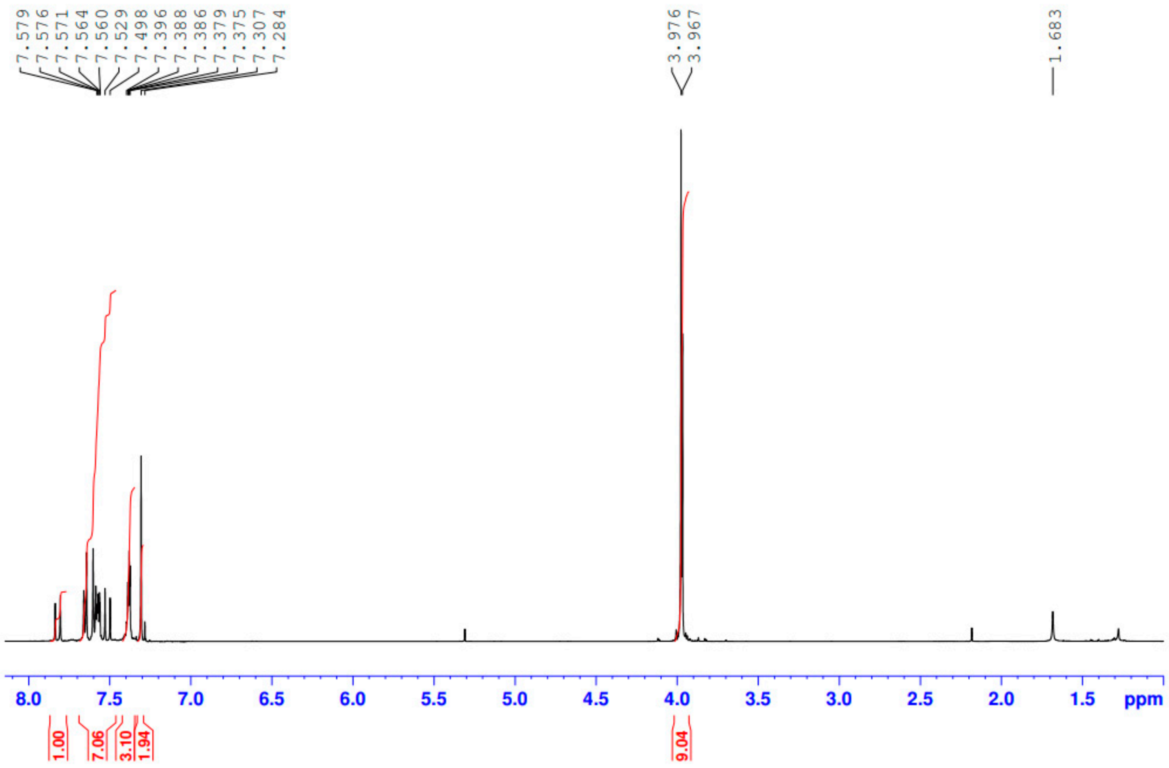

^{13}C-NMR of **36a** (125 MHz, DMSO-d6)

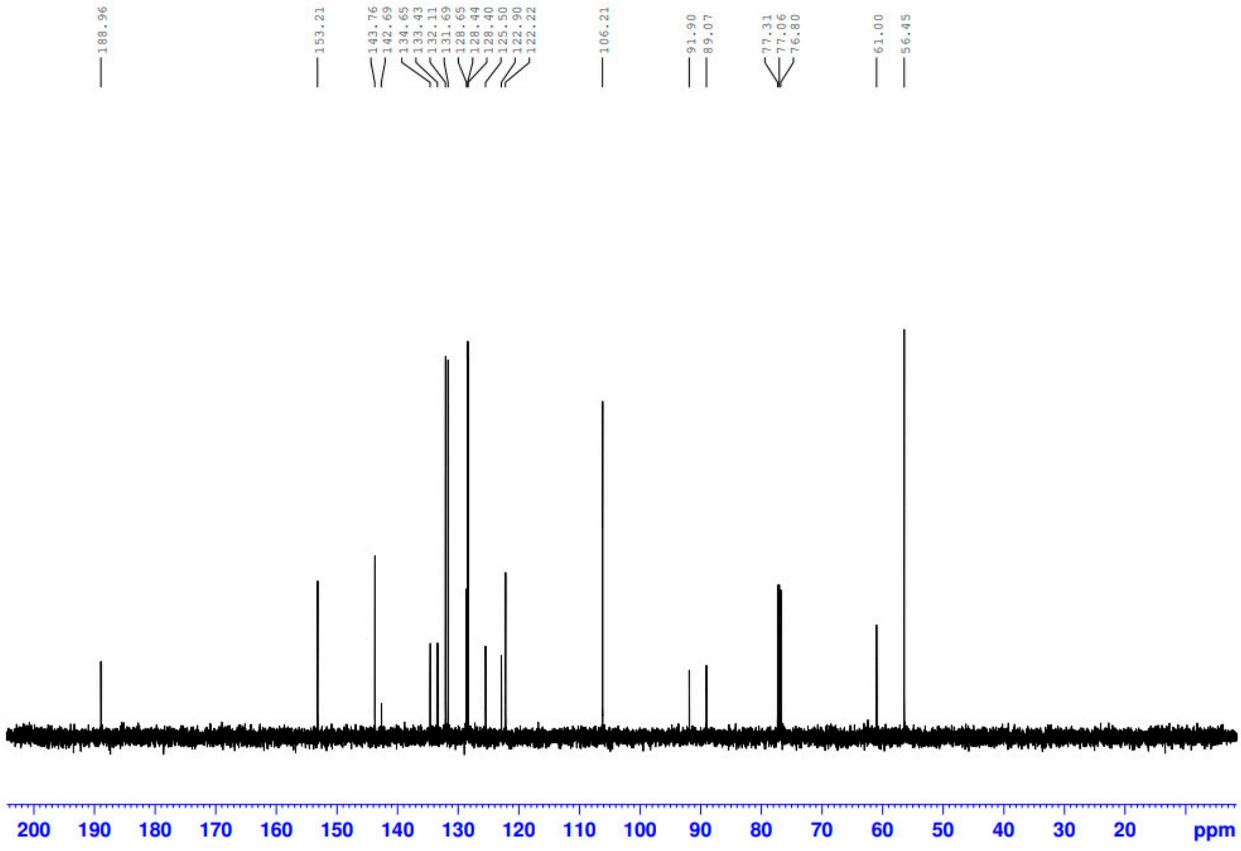

^{1}H-NMR of **37a** (500 MHz, DMSO-d$_6$)

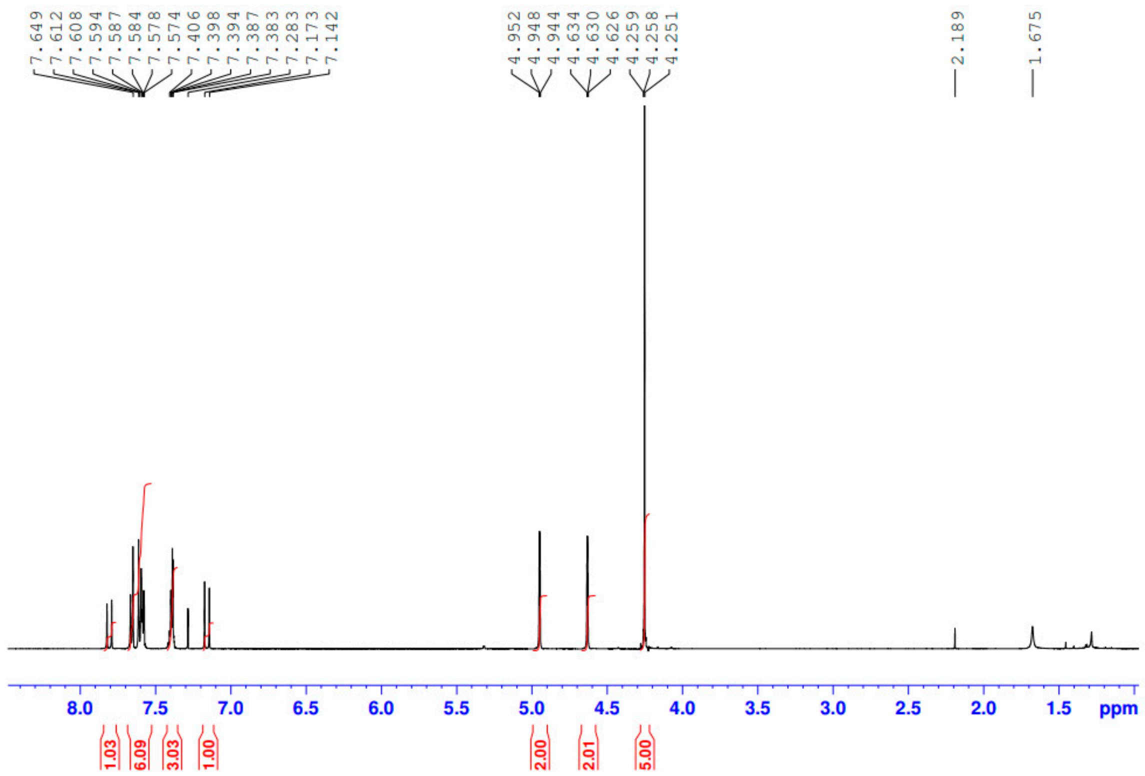

^{13}C-NMR of **37a** (125 MHz, DMSO-d$_6$)

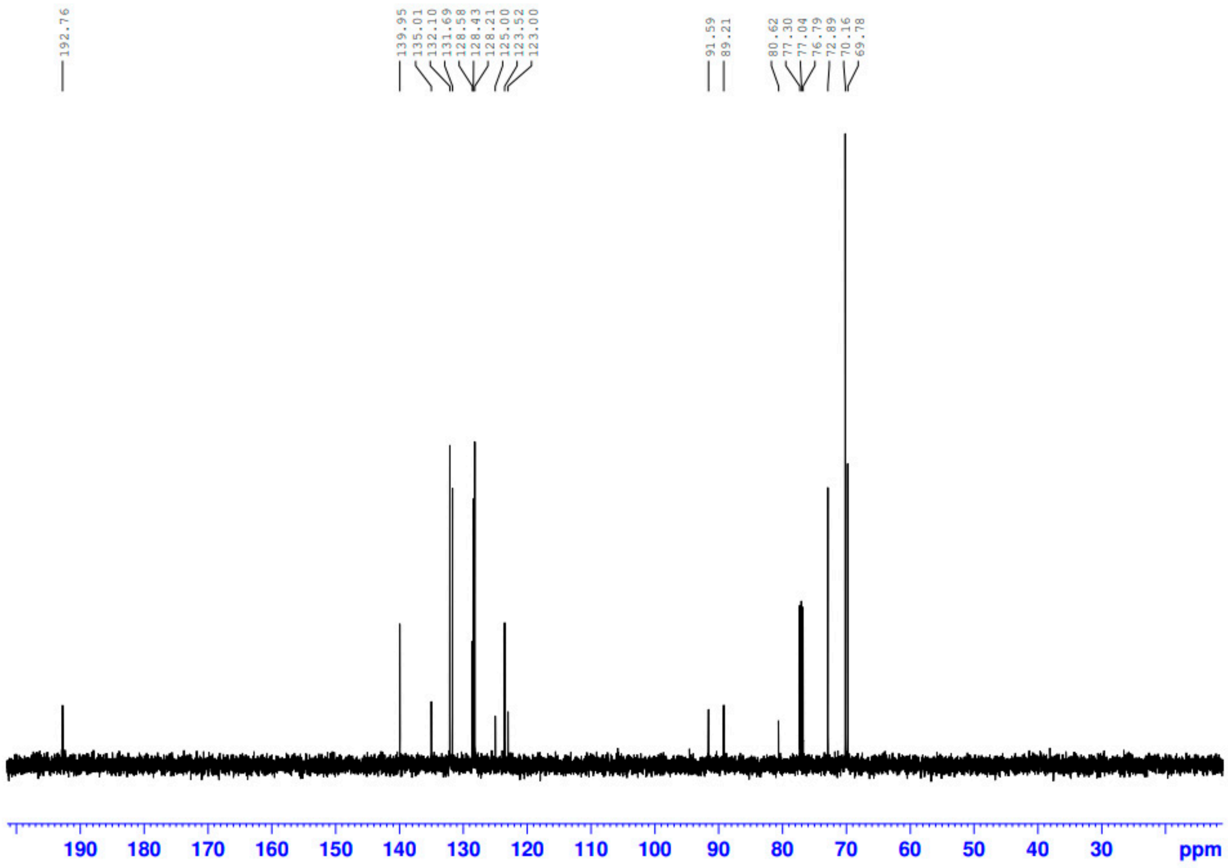

2 Copies of HRMS spectra

2.1 HRMS spectra of the novel alkyne-tethered vindoline hybrids

HRMS of **21**

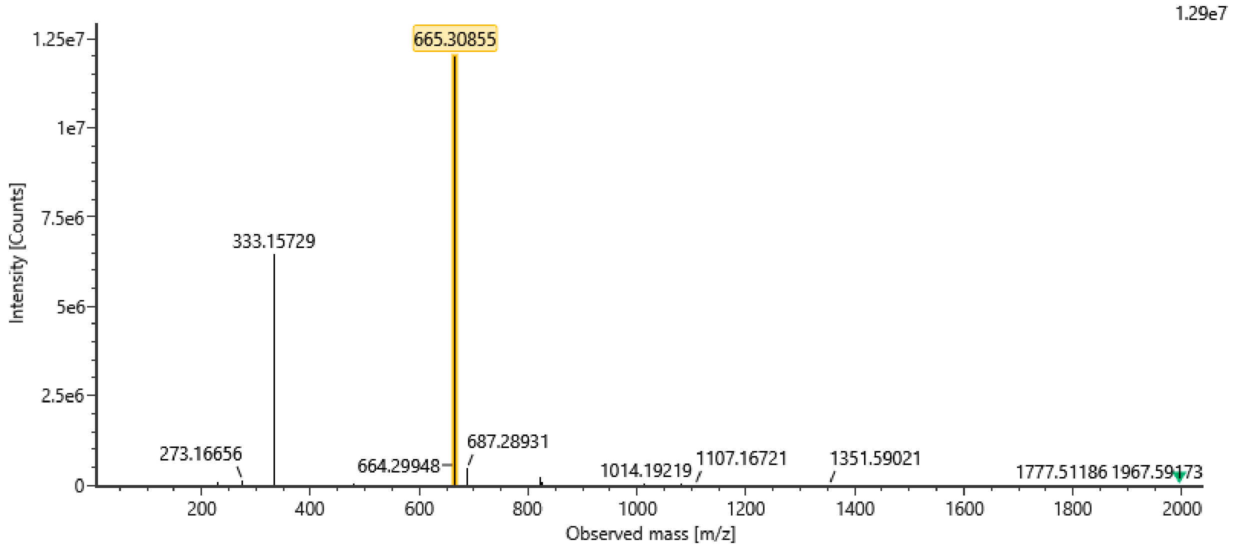

HRMS of **22**

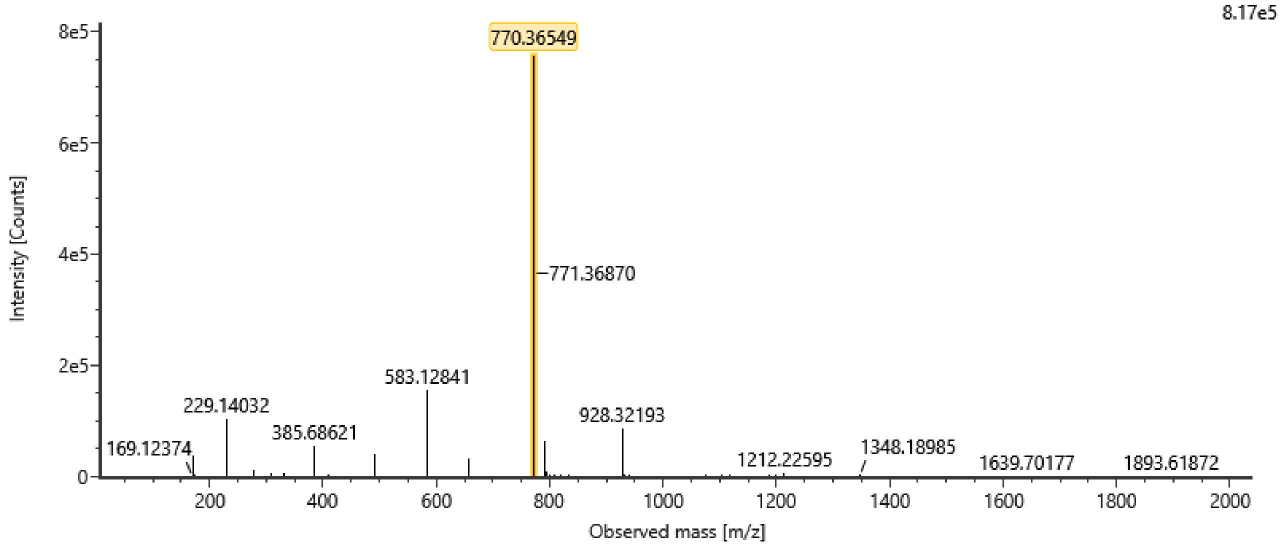

HRMS of **23**

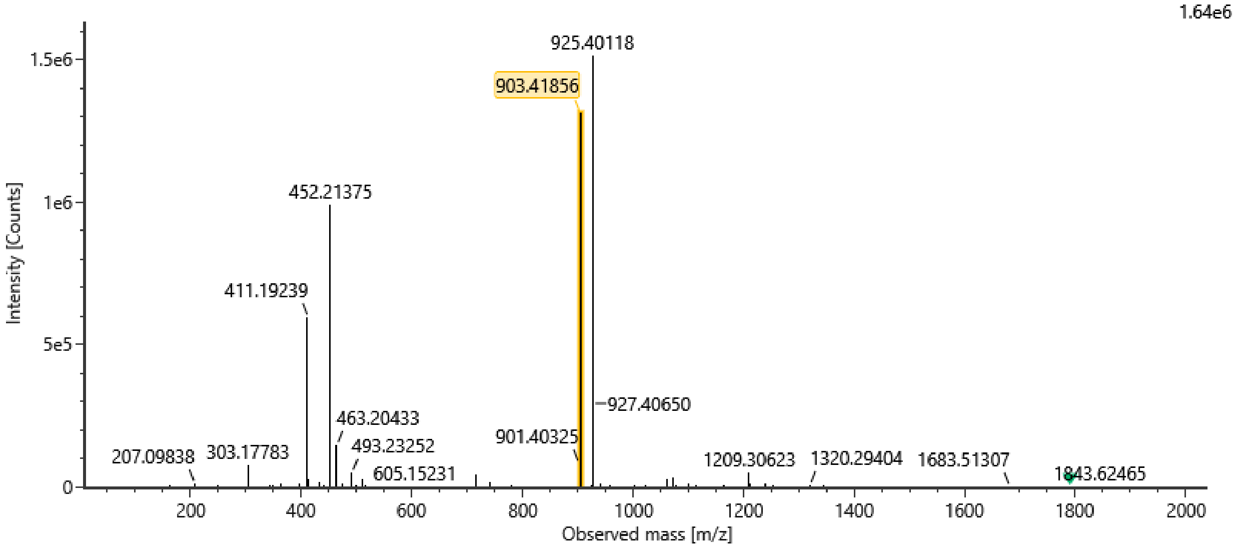

HRMS of **24**

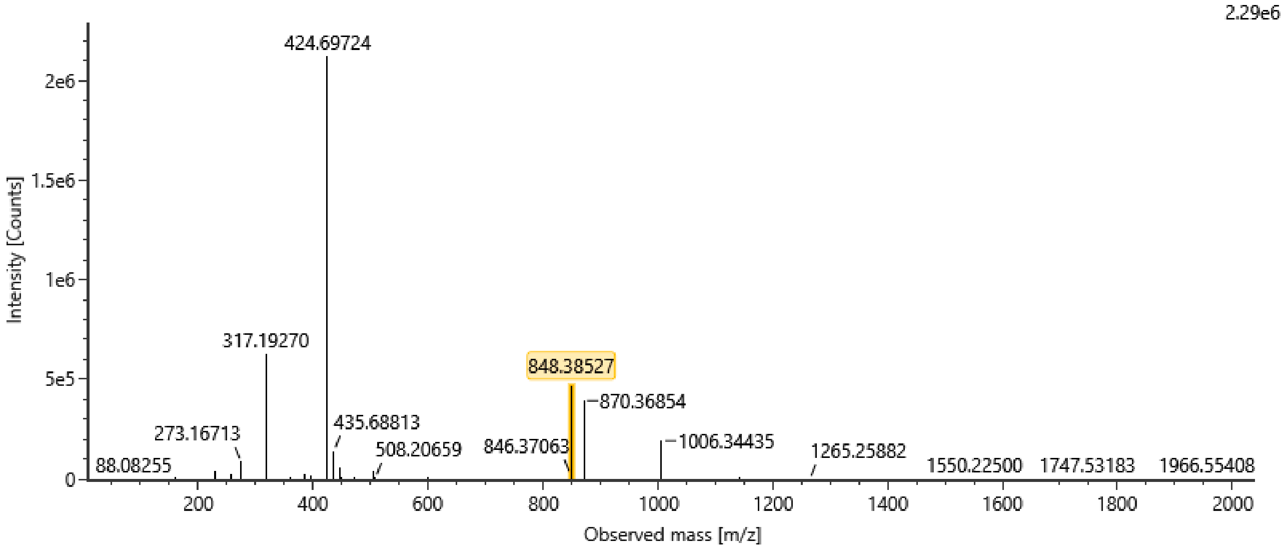

HRMS of **28**

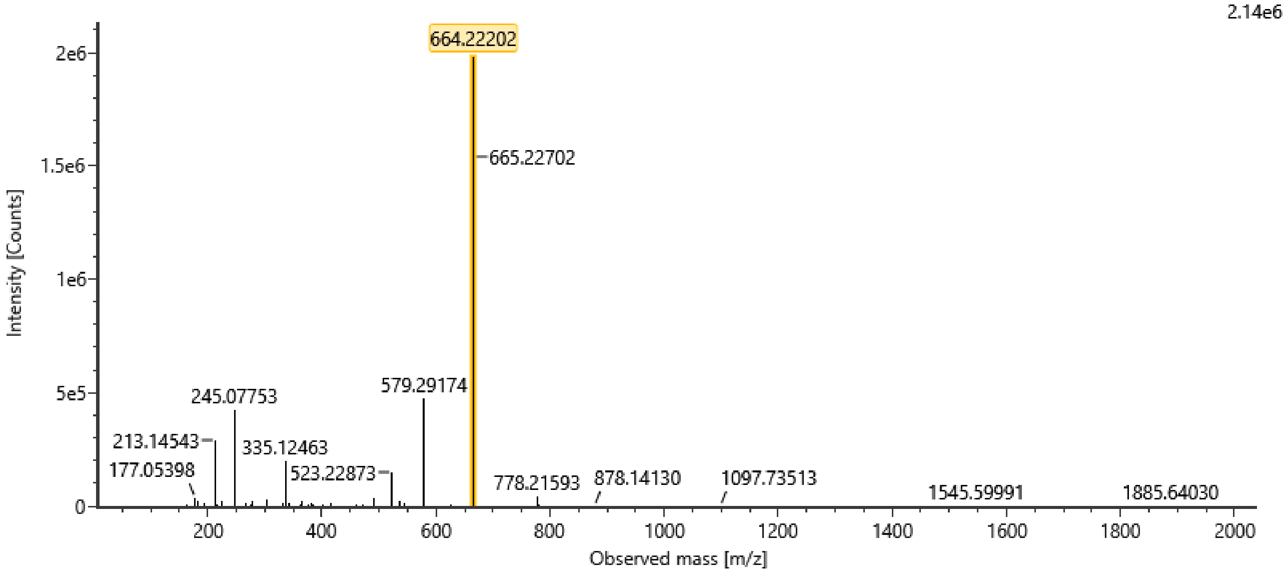

HRMS of **29**

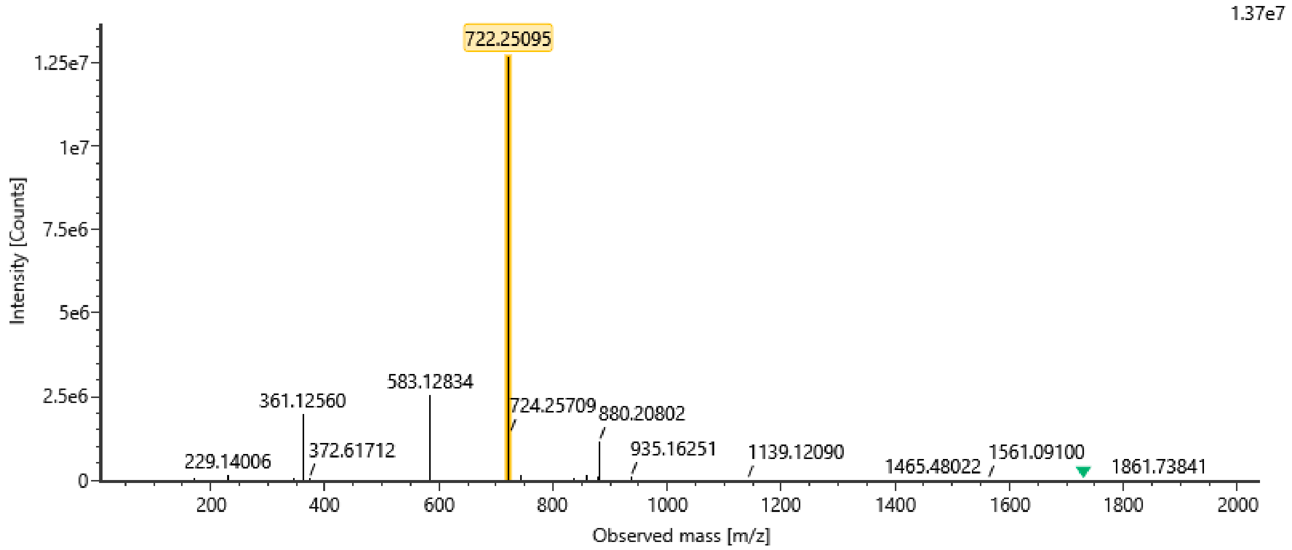

HRMS of **36**

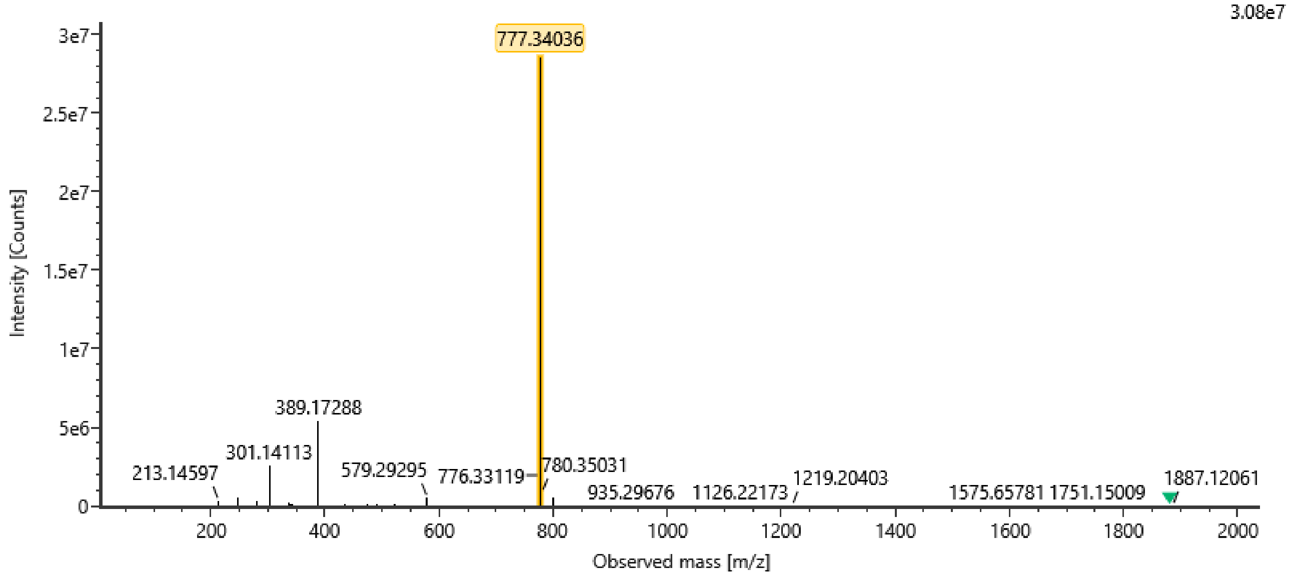

HRMS of **36a**

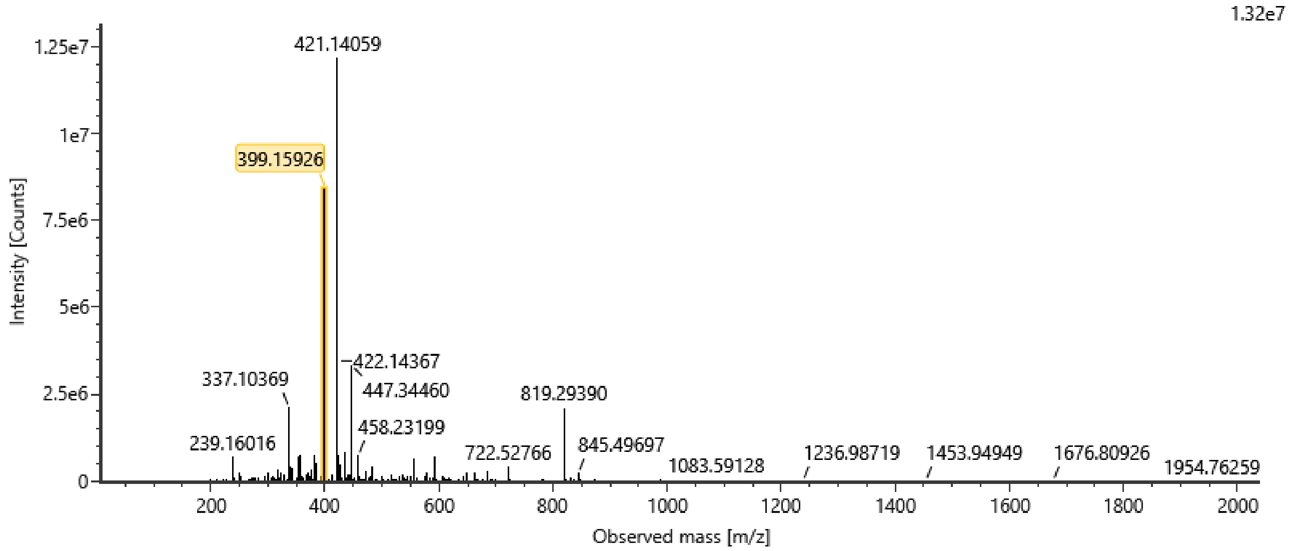

HRMS of **37**

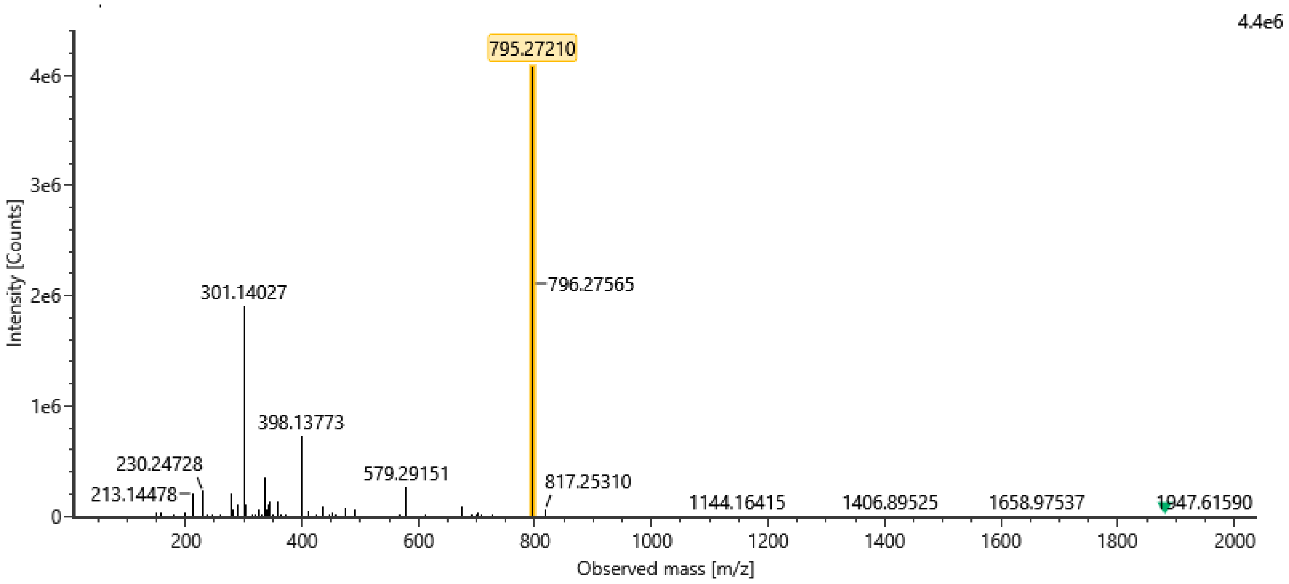

HRMS of **37a**

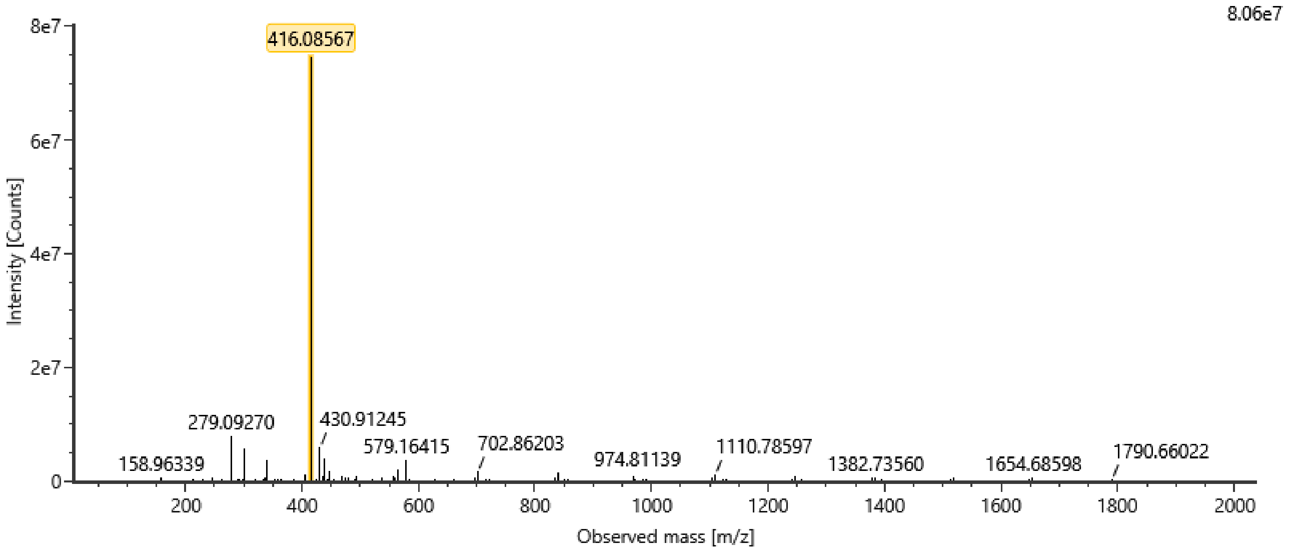

Disclaimer/Publisher's Note: The statements, opinions and data contained in all publications are solely those of the individual author(s) and contributor(s) and not of MDPI and/or the editor(s). MDPI and/or the editor(s) disclaim responsibility for any injury to people or property resulting from any ideas, methods, instructions or products referred to in the content.
